# The Multifaceted Mechanistic Actions of Antimicrobial Nanoformulations: Overcoming Resistance and Enhancing Efficacy

**DOI:** 10.3390/pharmaceutics18040423

**Published:** 2026-03-30

**Authors:** Renuka Gudepu, Ramadevi Kyatham, Nirmala Devi Ediga, Geetha Penta, Raju Bathula, Mohammed Mujahid Alam, Mounika Sarvepalli, Jayarambabu Naradala, Vikram Godishala, Swati Dahariya, Aditya Velidandi

**Affiliations:** 1 Department of Microbiology, Pingle Government College for Women (A), Warangal 506370, India; renumanduva@gmail.com; 2 School of Pharmacy, Anurag University, Hyderabad 500088, India; ramadevipharmacy@anurag.edu.in; 3 Department of Humanities and Basic Sciences (Physics), G. Pulla Reddy Engineering College (Autonomous), Kurnool 518007, India; nirmala.bs@gprec.ac.in; 4 Department of Physics, Sri Venkateswara College of Engineering, Tirupathi 517520, India; geetha.pt@svce.edu.in; 5 Santoshimata College of Pharmacy, Nasik 423403, India; dr.rajubathula@gmail.com; 6 Department of Chemistry, College of Science, King Khalid University, Abha 61421, Saudi Arabia; malm@kku.edu.sa; 7 School of Life Sciences, B.S. Abdur Rahman Crescent Institute of Science and Technology, Chennai 600048, India; mounika@crescent.education; 8 Department of Physics, National Institute of Technology, Warangal 506004, India; njayarambabu@gmail.com; 9 Department of Biotechnology, Vaagdevi Degree and P.G. College, Warangal 506001, India; vikramgodishala@gmail.com; 10 Department of Biochemistry, School of Life Sciences, University of Hyderabad, Hyderabad 500046, India; swati@uohyd.ac.in

**Keywords:** antimicrobial resistance, biofilm eradication, multifaceted mechanisms, nanoformulations, nanotoxicology, targeted drug delivery

## Abstract

Antimicrobial resistance represents one of the most formidable global health crises of the 21st century, driven by the diminishing efficacy of conventional antibiotics due to bacterial adaptation and biofilm formation. In response, antimicrobial nanoformulations have emerged as a transformative therapeutic paradigm, offering multifaceted and innovative mechanisms to combat resistant pathogens. This comprehensive review delineates the broad scope and distinct novelty of nano-enabled antimicrobial strategies, moving beyond the single-target limitations of traditional drugs. We systematically explore the diverse architectural classes of nanoformulations—including metallic, polymeric, and self-assembling nanostructures—and elucidate their unique mechanistic actions. These encompass (1) physical disruption of microbial membranes via electrostatic interactions; (2) catalytic generation of reactive oxygen and nitrogen species to induce an ‘oxidative storm’; (3) intracellular sabotage of essential metabolic pathways; (4) the ‘Trojan horse’ strategy for enhanced drug delivery and bioavailability; (5) efflux pump bypass to counteract a major resistance mechanism; (6) penetration and eradication of resilient biofilms; and (7) disarming pathogens through quorum sensing and virulence inhibition. Furthermore, this review highlights the immunomodulatory potential of nanoformulations; their activity beyond bacteria against fungi, viruses, and parasites; and the critical role of the nano-bio interface defined by surface physicochemistry. We also address the translational pathway, considering challenges in nanotoxicology, scalability, and regulatory approval, alongside the ecological impact and economic horizon of these technologies. This sector is projected to reach USD 5.4 to 8.96 billion by 2033 to 2034, with compound annual growth rates of 11 to 21% across antimicrobial nanomaterials, nanocoatings, and nanomedicine applications. By integrating insights from computational modeling and in silico design, this review underscores how nanoformulations leverage synergistic, multi-target approaches to overcome resistance, enhance therapeutic efficacy, and represent a significant leap forward in the future of infectious disease management. The novelty lies in the holistic and mechanistic synthesis of how nanotechnology is redefining antimicrobial warfare, offering a promising arsenal to avert a post-antibiotic era.

## 1. Introduction

### 1.1. The Crisis of Antimicrobial Resistance

The global landscape of antimicrobial resistance presents a complex and escalating public health challenge, characterized by significant epidemiological shifts and alarming statistics. The literature underscores the multifaceted nature of antimicrobial resistance, highlighting its mechanisms, prevalence, and far-reaching effects across different regions and healthcare settings [1].

Epidemiologically, antimicrobial resistance is increasingly recognized as a pervasive threat that transcends geographical boundaries. Teng et al. [2] provide insight into the resistance mechanisms in *Escherichia coli* isolates in Taiwan, where resistant strains were identified at 119 sites in 48 rivers through a nationwide surveillance program. Although the prevalence of these non-mcr colistin-resistant strains remained low and stable during the study period, their detection signifies the ongoing evolution of resistance mechanisms beyond well-characterized genes like mcr. Similarly, Jing et al. [3] conducted a comprehensive genomic epidemiology analysis of *Morganella* species globally, revealing the widespread distribution and evolution of resistance genes across different regions, emphasizing the importance of genomic surveillance in understanding antimicrobial resistances epidemiology.

Antimicrobial resistance in India is a significant public health challenge, characterized by high prevalence and mortality rates. The misuse of antibiotics, over-the-counter availability, and self-medication are key factors exacerbating this crisis. The prevalence of multidrug-resistant organisms is notably high in both community and healthcare settings, leading to increased morbidity and mortality. In a study [4] conducted at AIIMS Rishikesh, 1106 multidrug-resistant organisms were identified among 820 patients, with *Klebsiella pneumoniae*, *Acinetobacter baumannii*, and *E. coli* being the most common pathogens. The prevalence of multidrug-resistant organisms varied significantly across departments, with General Medicine and Surgery reporting the highest burden. A study [5] on community-onset infections found that 50% of bacterial isolates were multidrug-resistant, with a significant association between recent antibiotic use and the development of resistance. *A. baumannii* is responsible for 22.3% of hospital-acquired pneumonia cases in India, with carbapenem resistance rates exceeding 70% [6]. Enterobacteriaceae, particularly *K. pneumoniae*, are frequently isolated, with a high prevalence of extended-spectrum beta-lactamase producers [7]. The overall mortality rate among patients with multidrug-resistant organisms infections at AIIMS Rishikesh was 25.9% [4]. Another study reported a mortality rate of 13.1% among patients with multidrug-resistant infections, with significantly higher mortality associated with multidrug-resistant and extensively drug resistant strains of *E. coli*, *K. pneumoniae*, and *A. baumannii* [8].

In summary, the epidemiology of antimicrobial resistance is characterized by a rising prevalence of resistant pathogens across diverse settings, with significant numbers and statistics illustrating its global impact. The evidence points to a steady increase in resistance genes, regional variability in resistance patterns, and profound health and economic consequences. Addressing this crisis requires a comprehensive understanding of resistance mechanisms, robust surveillance systems, and effective policy interventions to prevent a future where antimicrobial agents become ineffective against common infections. The literature collectively emphasizes that without urgent and sustained action, the burden of antimicrobial resistance will continue to grow, threatening global health security and economic stability.

### 1.2. The Limitations of Conventional Antibiotics: A Single-Target Paradigm

The limitations of conventional antibiotics in combating bacterial infections are increasingly evident, primarily due to their propensity to foster resistance and their ineffectiveness against complex bacterial communities such as biofilms. These challenges are rooted in the fundamental mechanisms of how traditional antibiotics function and the biological defenses employed by bacteria, which collectively contribute to the rapid development of resistance.

**Figure 1:**
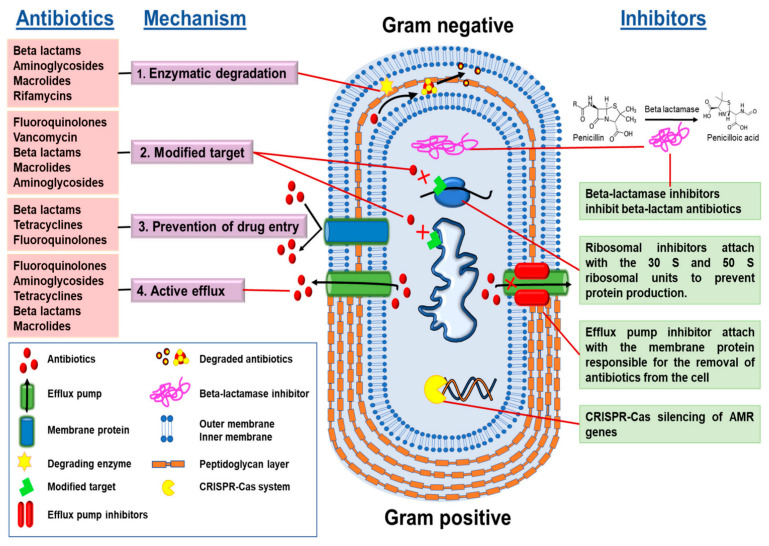
Classes of antibiotics, mechanism of action, and their respective inhibitors. Reproduced from [9].

**Figure 2:**
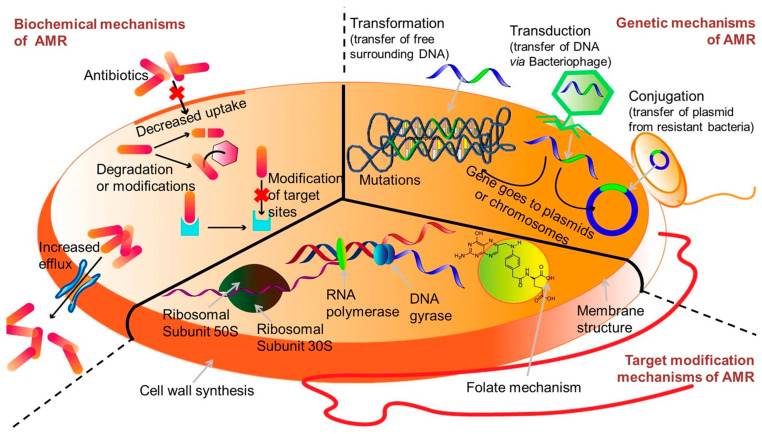
Schematic representation of differentiating the mechanisms of antibiotic action from those of antibiotic resistance, as they occur at the cellular and subcellular levels of the bacterial cell. Reproduced from [1].

One of the core issues with conventional antibiotics is their reliance on single-target mechanisms (Figure 1) [9]. Most traditional drugs are designed to interfere with specific bacterial processes, such as cell wall synthesis, protein synthesis, or DNA replication [10]. While initially effective, this narrow targeting creates a vulnerability: bacteria can quickly develop resistance through mutations or acquisition of resistance genes that alter or bypass the targeted pathway (Figure 2). The small number of targets in these antibiotics means that a single genetic change can render the drug ineffective, facilitating the rapid emergence of resistant strains [11].

Furthermore, the efficacy of antibiotics is often compromised by their inability to reach effective concentrations at the site of infection. Many antibiotics exhibit poor penetration into biofilms, which are structured communities of bacteria embedded within a self-produced extracellular matrix [12]. Biofilms serve as a formidable barrier, impeding the diffusion of antibiotics and creating microenvironments where bacteria can survive sub-lethal drug concentrations. This limited penetration not only diminishes the immediate bactericidal effect but also promotes the selection of resistant bacteria within the biofilm, further complicating treatment [12].

The issue of low antibiotic concentrations at infection sites is compounded by the pharmacokinetic limitations of many traditional drugs. Achieving and maintaining therapeutic levels without causing toxicity is challenging, often resulting in sub-inhibitory concentrations that favor the development of resistance [10]. Sub-lethal exposure to antibiotics can induce stress responses in bacteria, leading to the activation of resistance mechanisms such as efflux pumps, enzymatic degradation, and target modification [13]. These adaptive responses enable bacteria to survive subsequent antibiotic exposure, accelerating the resistance process.

Biofilm-associated infections exemplify the limitations of conventional antibiotics. Bacteria within biofilms exhibit heightened resistance compared to planktonic counterparts, owing to multiple mechanisms. These include restricted antibiotic penetration, altered microenvironmental conditions (such as pH and oxygen levels), and the presence of persisted cells—dormant bacteria that are inherently tolerant to antibiotics [12,14]. The resistance conferred by biofilm formation is particularly problematic because it leads to chronic infections that are difficult to eradicate with standard antibiotic therapy. In addition to these biological barriers, the mechanisms of resistance are often multifaceted. Bacteria employ efflux pumps to actively expel antibiotics, modify drug targets, produce enzymes that degrade antibiotics, and alter membrane permeability [13]. These strategies are often induced or enhanced in response to antibiotic exposure, especially when drugs are used at low concentrations or for insufficient durations. The presence of biofilms further exacerbates these issues by providing a protective niche where resistance genes can be exchanged among bacteria, facilitating horizontal gene transfer [14].

The limitations of traditional antibiotics have prompted the exploration of alternative strategies [10]. For instance, antimicrobial peptides and nanotechnology-based delivery systems are being investigated for their potential to overcome biofilm barriers and reduce resistance development [15,16,17]. These approaches aim to target multiple bacterial components simultaneously or enhance drug delivery to infection sites, thereby reducing the likelihood of resistance emergence. The use of nanobiotics, in particular, offers promising avenues for delivering drugs effectively while minimizing resistance, given their ability to penetrate biofilms and deliver high local drug concentrations [1,17].

In summary, the inherent design of conventional antibiotics—primarily their single-target action, limited biofilm penetration, and susceptibility to low-dose exposure—contributes significantly to the rapid development of resistance. The complex defense mechanisms of bacteria, especially within biofilms, further diminish the efficacy of traditional drugs. Addressing these limitations requires innovative approaches that can target multiple bacterial pathways, improve drug delivery, and circumvent resistance mechanisms. The ongoing research into antimicrobial peptides, nanotechnology, and other novel therapies reflects a concerted effort to overcome the shortcomings of conventional antibiotics and combat the escalating threat of antimicrobial resistance.

### 1.3. Nanotechnology as a Disruptive Therapeutic Platform

The advent of nanotechnology has heralded a transformative era in therapeutic strategies, particularly in the fight against microbial infections. Traditionally, antimicrobial therapies have relied heavily on molecular agents such as antibiotics, which have become increasingly challenged by the rise of resistant bacterial strains [10]. This paradigm shift towards nanoformulations signifies a move from conventional molecular warfare to particulate-based interventions, offering novel mechanisms to disrupt microbial viability and combat resistance.

Nanoparticles have emerged as versatile tools in drug delivery systems, capable of enhancing the efficacy and specificity of antimicrobial agents [17]. As noted in recent studies, nanoparticles can be employed to disrupt bacterial membranes directly, thereby overcoming some of the limitations associated with traditional antibiotics, such as poor penetration and rapid degradation [10]. This approach leverages the unique physicochemical properties of nanomaterials, including their high surface area-to-volume ratio and tunable surface functionalities, which facilitate targeted interactions with bacterial cells.

The concept of nanoformulations as a paradigm shift is further supported by their potential to address biofilm-associated infections. Biofilms pose a significant challenge in clinical settings due to their inherent resistance to conventional antibiotics. Nanotechnology-based strategies have demonstrated promise in disrupting biofilm architecture and targeting embedded bacteria, thereby restoring susceptibility to antimicrobial agents [18]. These multifunctional nanocarriers can deliver therapeutic payloads directly into biofilms, enhancing drug penetration and efficacy.

Beyond direct antimicrobial activity, nanotechnology also enables innovative delivery platforms for alternative therapies such as bacteriophages and RNA-based treatments. Multifunctional nanocarriers have been developed to facilitate bacteriophage delivery, which offers a promising alternative to antibiotics, especially against resistant strains [19]. These nanocarriers can protect phages from degradation and improve their targeting efficiency. Similarly, RNA nanoparticles have been utilized to deliver therapeutic molecules, representing a shift towards personalized and noninvasive antimicrobial therapies [20]. The modularity of RNA platforms allows for customizable delivery systems that can be tailored to specific microbial targets.

The integration of nanotechnology into antimicrobial strategies aligns with broader scientific trends emphasizing disruptive innovations. As highlighted in recent analyses, such paradigm shifts have the potential to redefine therapeutic landscapes over decades, moving from traditional molecular approaches to particulate and nanostructured interventions [21]. This transition is particularly relevant in the context of rising antibiotic resistance, where conventional drugs are losing efficacy, and novel approaches are urgently needed.

In summary, nanotechnology represents a disruptive platform in therapeutics, particularly in the realm of antimicrobial strategies. The shift from molecular to particulate warfare offers innovative avenues for disrupting bacterial membranes, biofilms, and resistance mechanisms. As research continues to advance, nanoformulations are poised to redefine the landscape of infectious disease management, embodying a true paradigm shift in biomedical science. This evolution underscores the importance of integrating nanotechnological innovations into future therapeutic frameworks to address the pressing challenges posed by resistant microbes and emerging infectious threats.

## 2. Classification and Mechanisms Antimicrobial Action

### 2.1. The Architectural Toolkit: Classes of Antimicrobial Nanoformulations

**Figure 3:**
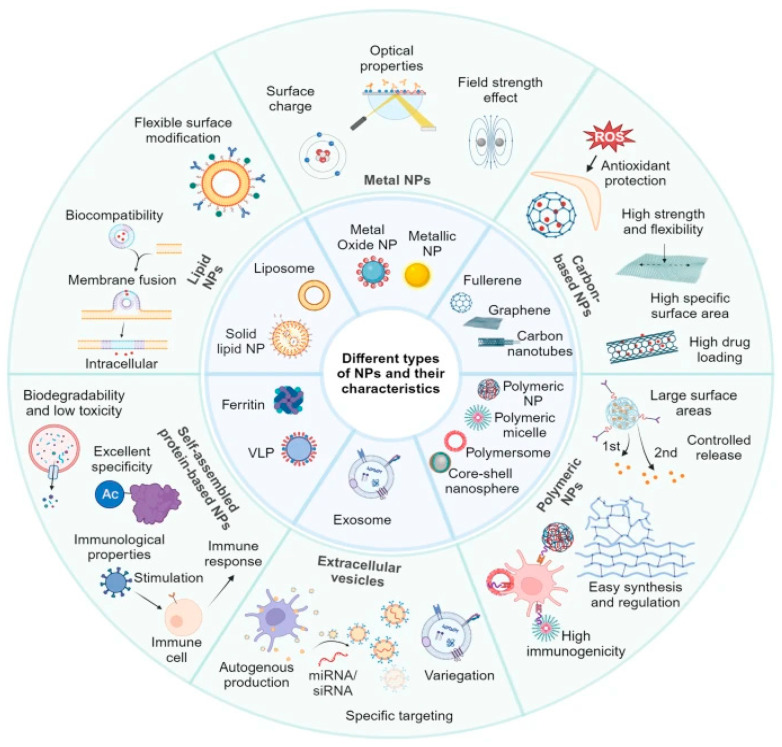
Six common nanomaterials and their characteristics. Reproduced from [17].

The architectural toolkit of antimicrobial nanoformulations encompasses a diverse array of nanomaterials and nanostructures designed to combat microbial pathogens, particularly in the face of rising antimicrobial resistance (Figure 3). Recent literature highlighted the significance of various classes of nanomaterials, their mechanisms of action, and their potential applications in infection control and wound healing.

Nanomedicine has revolutionized drug delivery strategies, with nanomaterials serving as vehicles to enhance the efficacy and targeting of antimicrobial agents. Nanoparticles can improve bioavailability, facilitate targeted delivery, and enable controlled release of therapeutic agents [22]. These features are especially valuable in treating resistant infections, where conventional antibiotics often fail due to poor penetration or rapid degradation. The integration of nanomaterials into drug delivery systems also opens avenues for combining multiple antimicrobial agents, thereby exploiting synergistic effects and reducing the likelihood of resistance development.

**Figure 4:**
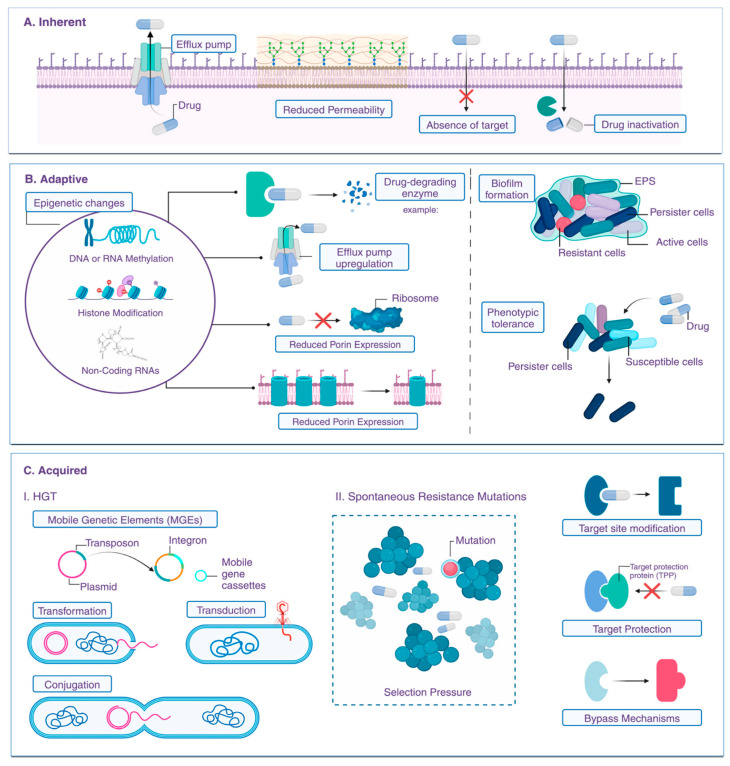
Mechanisms of antimicrobial resistance in *P. aeruginosa*: (**C**) Acquired, (**A**) intrinsic, and (**B**) adaptive pathways. Reproduced from [24].

The challenge of antimicrobial resistance, particularly in pathogens like *P. aeruginosa*, underscored the need for innovative approaches. Resistance mechanisms such as efflux pumps, enzymatic degradation, and biofilm formation pose significant hurdles to traditional antibiotics (Figure 4) [23]. Nanoparticles, with their multifaceted mechanisms, offer a promising alternative. Advanced nanomaterials, including hybrid and multifunctional nanoparticles, are being developed to address these challenges. For example, the use of nanostructured antibiotics and inorganic nanomaterials has shown potential in overcoming bacterial defenses and reducing resistance [24]. The expanding toolbox against antimicrobial resistance emphasizes the importance of designing nanostructures with specific architectures to maximize antimicrobial efficacy. Synthetic strategies that incorporate natural antimicrobial peptides, inorganic nanoparticles, and organic nanocarriers enable the creation of multifunctional platforms capable of targeting resistant bacteria effectively [25]. These architectures can be tailored to optimize interactions with bacterial membranes, biofilm disruption, and delivery of antimicrobial agents, thereby enhancing therapeutic outcomes.

In summary, the architectural toolkit of antimicrobial nanoformulations comprises a broad spectrum of nanomaterials, including self-assembling peptides, inorganic nanoparticles like silver and gallium-based structures, and nanostructured antibiotics. Their diverse mechanisms of action—ranging from membrane disruption to biofilm inhibition—highlight their potential in addressing the global challenge of antimicrobial resistance. The integration of nanotechnology into antimicrobial strategies offers promising avenues for developing more effective, targeted, and durable therapies, ultimately transforming the landscape of infection control and wound management.

### 2.2. The First Line of Attack: Physical Membrane Disruption

The initial line of attack employed by many nanoformulations against microbial pathogens predominantly involves the disruption of the microbial membrane through physical and electrostatic interactions. This broad-spectrum mechanism is fundamental to the efficacy of various nanomaterials and antimicrobial peptides, which target the integrity of the microbial envelope, leading to cell death.

One of the most extensively studied classes of antimicrobial agents that utilize membrane disruption are antimicrobial peptides. Buforin II, a well-characterized antimicrobial peptide, exemplifies this mechanism by exerting antibacterial activity across a wide range of bacteria [26]. Its mode of action involves penetrating the microbial membrane and destabilizing it, which results in leakage of cellular contents and eventual cell death. This process underscores the importance of physical interactions between the peptide and the microbial envelope, which are facilitated by electrostatic attractions between the cationic peptides and the negatively charged bacterial membranes. Similarly, synthetic cationic polymers such as hyper-branched poly-L-lysine demonstrated a comparable mechanism. These polymers interact electrostatically with microbial membranes, leading to physical disruption [27]. The interaction involves the attraction of positively charged groups on the polymer to negatively charged components of the bacterial cell wall, which destabilizes the membrane structure. Despite the effectiveness of this approach, some studies suggest that the precise mechanisms may involve additional processes such as oxidative stress or protein dysfunction, but membrane disruption remains a central theme.

Nanoparticles, particularly metallic ones like silver and zinc oxide, have gained prominence for their broad-spectrum antimicrobial activity, primarily through membrane disruption facilitated by electrostatic interactions. Silver nanoparticles, for instance, have been shown to cause damage to bacterial membranes, leading to increased permeability and cell lysis [28]. The interaction of silver nanoparticles with the bacterial envelope induces oxidative stress, protein dysfunction, and DNA damage, but the initial physical contact and membrane perturbation are critical steps in their bactericidal action [28,29]. The surface charge of silver nanoparticles plays a pivotal role in their ability to adhere to and disrupt microbial membranes, emphasizing the importance of electrostatic forces.

The importance of membrane disruption as a first line of attack is further supported by studies on antimicrobial textiles and coatings. These materials incorporate nanomaterials that physically interact with microbial membranes, leading to their inactivation. For instance, antimicrobial textiles utilize electrostatic interactions and membrane disruption mechanisms to prevent microbial growth [30]. The physical contact between nanomaterials and microbes results in membrane destabilization, which is crucial for achieving rapid and broad-spectrum antimicrobial effects. In addition to metallic and polymeric nanomaterials, natural and synthetic surface coatings also employ membrane disruption as a key mechanism. These coatings, which include metals, polymers, and biopolymers, exert their antiviral and antimicrobial effects partly through physical interactions that compromise the integrity of microbial envelopes [31]. The disruption of the membrane barrier not only kills the microbes but also prevents their proliferation, making this mechanism highly effective for surface sterilization and infection control. Recent advancements in non-thermal plasma technologies further illustrate the significance of physical membrane disruption. Non-thermal plasma-treated materials, such as chitosan, exhibit enhanced antibacterial activity through mechanisms that include membrane damage [32]. The plasma treatment modifies the physical properties of the material, enabling it to interact more effectively with microbial membranes and induce disruption.

In summary, the first line of microbial attack by nanoformulations predominantly involves physical and electrostatic interactions that lead to membrane disruption. This mechanism is central to the broad-spectrum activity observed in antimicrobial peptides, cationic polymers, metallic nanoparticles, and nanostructured coatings. The ability of these agents to target and destabilize the microbial envelope underscores their potential in combating resistant pathogens and developing effective antimicrobial strategies. The convergence of physical disruption and electrostatic attraction forms a robust foundation for the design of next-generation nanomaterials aimed at microbial inactivation.

### 2.3. The Oxidative Storm: Catalytic Generation of Reactive Species

The mechanistic intricacies of reactive oxygen species and reactive nitrogen species generation by nanoformulations have garnered significant attention due to their pivotal role in microbial oxidative damage and potential therapeutic applications. The current literature underscored the catalytic processes underpinning reactive oxygen species and reactive nitrogen species production, their biological implications, and the innovative nanotechnological strategies employed to harness these reactive species for antimicrobial purposes.

Nanoformulations, particularly nanozymes and nanomaterials, serve as catalytic platforms that facilitate the continuous generation of reactive oxygen species and reactive nitrogen species, thereby inducing oxidative stress within microbial cells. For instance, nanoplatforms in sepsis management have been shown to produce reactive oxygen species directly capable of damaging bacterial components, highlighting the potential of nanomaterials to generate reactive species in a controlled and targeted manner [33]. Similarly, ultrasound-driven manganese oxide nanocatalysts exemplify how catalytic loops can be exploited to sustain reactive species production, culminating in a potent oxidative storm within the tumor microenvironment, which can be extrapolated to microbial contexts [34].

The catalytic mechanisms involve redox cycling and electron transfer processes intrinsic to nanomaterials. These processes facilitate the conversion of molecular oxygen and other substrates into reactive species such as hydroxyl radicals, superoxide anions, and singlet oxygen. The generation of these species is often enhanced by external stimuli like ultrasound or ultraviolet radiation, which activate the nanocatalysts and amplify reactive oxygen species and reactive nitrogen species production [34,35]. Such catalytic activity is crucial for overcoming microbial defenses, as the reactive species can indiscriminately damage vital cellular components. The oxidative damage inflicted by reactive oxygen species and reactive nitrogen species on microbial cells encompasses a broad spectrum of macromolecular targets. DNA, proteins, and lipids are particularly susceptible to oxidative modifications, leading to impaired cellular functions and eventual cell death. Excessive reactive oxygen species generation, whether through nanocatalytic activity or environmental stressors, results in lipid peroxidation, protein oxidation, and DNA strand breaks, thereby compromising microbial viability [36,37]. The destructive capacity of reactive oxygen species and reactive nitrogen species is further amplified in the presence of nanomaterials that can induce redox imbalances, intensifying oxidative stress within microbial cells [38].

Mechanistically, nanomaterials can induce oxidative stress by disrupting mitochondrial energy metabolism in microbes, activating signaling pathways that lead to increased reactive oxygen species production [39]. This disruption not only damages cellular components directly but also triggers inflammatory responses and cellular apoptosis. The ability of nanomaterials to catalyze the formation of reactive species is thus central to their antimicrobial efficacy, as it leads to a self-perpetuating cycle of oxidative damage. Furthermore, the generation of reactive species by nanomaterials is influenced by their physicochemical properties, such as surface area, composition, and catalytic activity. For example, nanomaterials like manganese oxide nanocatalysts exhibit a catalytic loop that sustains reactive oxygen species production, creating a continuous oxidative assault on microbial cells [34]. The design of such nanoplatforms aims to maximize reactive species generation while minimizing collateral damage to host tissues, emphasizing the importance of controlled catalytic processes.

Environmental factors and external stimuli also modulate reactive oxygen species and reactive nitrogen species production. Ultraviolet radiation, for instance, enhances reactive oxygen species generation, leading to oxidative stress when the balance tips towards excessive reactive species [35]. Similarly, exposure to heavy metals and other contaminants can induce reactive oxygen species formation, contributing to oxidative tissue damage and microbial killing [40]. These insights highlight the multifaceted nature reactive oxygen species and reactive nitrogen species generation mechanisms, which can be exploited or mitigated depending on therapeutic goals.

In summary, the catalytic generation of reactive oxygen species and reactive nitrogen species by nanoformulations involves complex mechanistic pathways centered around redox reactions and catalytic cycling. These reactive species induce oxidative damage to microbial macromolecules, leading to cell death and offering promising avenues for antimicrobial strategies. The ability to harness and control these processes through nanotechnology holds significant potential for combating resistant microbial strains and managing oxidative stress-related pathologies.

### 2.4. Intracellular Sabotage: Targeting Essential Metabolic Pathways

Intracellular sabotage of antimicrobial nanoformulations targeting essential metabolic pathways represents a promising yet complex strategy to combat pathogenic microorganisms. The mechanisms by which nanoformulations exert their antimicrobial effects after cellular entry primarily involve enzyme inhibition, protein denaturation, and nucleic acid damage, each disrupting vital metabolic processes within the pathogen.

One of the key mechanisms involves enzyme inhibition, which impairs critical enzymatic functions necessary for microbial survival and proliferation. For instance, certain nanomaterials are designed to interfere with enzymes involved in metabolic pathways, effectively halting essential biochemical reactions. Although specific enzyme targets are not detailed in the literature, the concept aligns with the broader understanding that disrupting enzyme activity can lead to metabolic collapse within the pathogen. The importance of targeting enzymes such as Cdc14 in fungi underscored the potential of enzyme inhibition as an antimicrobial strategy, as these enzymes are integral to cell cycle regulation and metabolic homeostasis [41].

Protein denaturation is another crucial mechanism through which nanoformulations exert antimicrobial effects intracellularly. Nanoparticles can induce structural alterations in microbial proteins, leading to loss of function and subsequent cell death. The ability of nanoparticles to denature proteins is linked to their physicochemical properties, such as surface charge and reactivity, which facilitate interactions with microbial proteins. This process can compromise the integrity of essential proteins involved in metabolism, signaling, and structural maintenance, thereby crippling the pathogen’s ability to sustain vital functions. The oxidative stress induced by nanotherapeutics further exacerbates protein denaturation, as reactive oxygen species can oxidize amino acid residues, leading to misfolding and aggregation of proteins [42].

Nucleic acid damage constitutes another pivotal mechanism, whereby nanoformulations induce direct or indirect harm to microbial DNA or RNA. This damage can manifest as strand breaks, base modifications, or cross-linking, ultimately impairing replication and transcription processes. The disruption of nucleic acids hampers the pathogen’s ability to produce essential proteins and enzymes, leading to cell death. Although specific studies on nucleic acid targeting are not explicitly detailed in the provided documents, the general principle is that nanoformulations capable of penetrating cellular compartments can access and damage genetic material, thereby sabotaging the pathogen’s genetic integrity [43]. The intracellular activity of antimicrobial nanoformulations is further complicated by the pathogen’s ability to evade host defenses and develop resistance. For example, the fusion of phagosomes with lysosomes is a critical step in host defense, and some pathogens have evolved mechanisms to inhibit this process, thereby avoiding intracellular destruction [43].

Nanoformulations that can bypass or disrupt such evasion strategies may enhance antimicrobial efficacy. The use of albumin-based nanoparticles, which offer biocompatibility and targeted delivery, exemplifies advances in designing nanoformulations capable of intracellular targeting [44]. Moreover, oxidative stress plays a significant role in the intracellular antimicrobial activity of nanotherapeutics. The generation of reactive oxygen species within infected cells can lead to oxidative damage of proteins, lipids, and nucleic acids, further impairing microbial metabolic pathways [42]. Antioxidant nanotherapeutic approaches aim to modulate this oxidative stress, either by enhancing reactive oxygen species production to kill pathogens or by protecting host cells from collateral damage [42]. In addition to direct microbial targeting, some nanomaterials are being explored for their ability to sensitize bacteria to conventional antibiotics or disrupt microbial defense mechanisms. For instance, certain phages encode antimicrobial proteins that can weaken bacterial defenses, making them more susceptible to nanoformulation-induced damage [45]. This combinatorial approach underscores the potential of intracellular sabotage strategies to overcome resistance and improve antimicrobial outcomes.

In summary, the intracellular sabotage of pathogens by antimicrobial nanoformulations involves multifaceted mechanisms targeting essential metabolic pathways. Enzyme inhibition disrupts critical biochemical reactions, protein denaturation impairs structural and functional proteins, and nucleic acid damage hampers genetic integrity. Advances in nanoparticle design, such as albumin-based platforms, enhance targeted delivery and intracellular access, amplifying these mechanisms. Understanding these processes is vital for developing effective nanotherapeutics capable of overcoming microbial resistance and achieving intracellular pathogen eradication.

### 2.5. The Trojan Horse Strategy: Enhanced Drug Delivery and Bioavailability

The escalating threat of antimicrobial resistance has intensified the search for innovative drug delivery approaches that enhance the efficacy of conventional antibiotics. Among these, nanotechnology-based delivery systems have emerged as promising tools, leveraging the Trojan horse strategy to improve targeted delivery, protect antibiotics from degradation, and overcome solubility and stability issues [17]. This section discusses current insights into how nanoformulations facilitate the Trojan horse approach, enabling antibiotics to reach infection sites more effectively and circumvent bacterial defenses.

**Figure 5:**
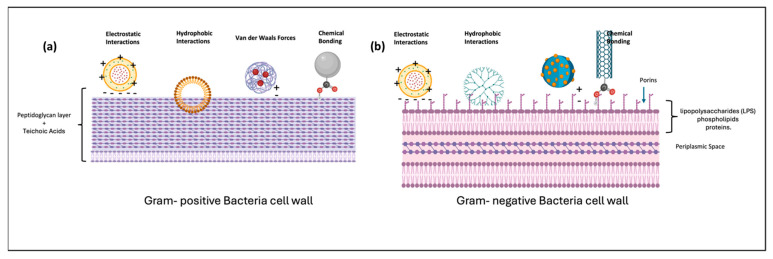
Interactions of nanoantibiotics with (**a**) Gram-positive and (**b**) Gram-negative bacterial membranes. Reproduced from [46].

Nanomaterials possess unique physicochemical properties that enable multiple bactericidal pathways, including direct bacterial membrane disruption and enhanced drug delivery [17]. The Trojan horse strategy involves encapsulating or associating antibiotics within nanocarriers, which can traverse biological barriers and deliver their payload directly to the infection site. This approach not only enhances the local concentration of antibiotics but also minimizes systemic toxicity, a common concern with high-dose therapies [46]. For instance, nanoantibiotics utilize nanocarriers to encapsulate conventional drugs, shielding them from premature degradation and ensuring their stability until reaching the target bacteria (Figure 5) [47]. One of the primary challenges in antibiotic therapy is poor solubility and stability, which limit drug bioavailability and efficacy. Nanotechnology offers solutions by improving these parameters through various nanocarrier systems such as lipid-based nanoparticles, polymeric nanoparticles, and other nanostructures [48]. Lipid-based nanoparticles, in particular, have demonstrated significant potential in enhancing drug solubility and stability, facilitating sustained release, and protecting antibiotics from enzymatic degradation [49]. These nanocarriers can be engineered to respond to specific stimuli at the infection site, further refining targeted delivery.

The ability of nanocarriers to bypass biological barriers is crucial for delivering antibiotics to otherwise inaccessible sites, such as ocular tissues or deep-seated infections [48]. For example, nanotechnology-based drug delivery systems have been designed to overcome ocular barriers, ensuring that therapeutic concentrations of antibiotics reach the target tissue [48]. Similarly, topical delivery strategies utilizing nanocarriers can enhance drug penetration and retention at the infection site, thereby increasing therapeutic efficacy [50]. These strategies exemplify how nanocarriers can serve as Trojan horses, ferrying antibiotics past physiological barriers that typically hinder drug access. Furthermore, the concept of utilizing infected cells as Trojan horses has gained attention. Infected cells can internalize nanocarriers loaded with antibiotics, effectively transporting the drugs directly to intracellular bacteria [46]. This approach is particularly relevant for infections caused by intracellular pathogens, which are often resistant to conventional antibiotics due to limited cellular penetration. By exploiting the natural phagocytic pathways of infected cells, nanocarriers can deliver antibiotics precisely where they are needed, enhancing treatment outcomes.

Combining multiple antibiotics within nanocarriers is another promising avenue, as it can address bacterial resistance mechanisms and prevent the emergence of resistant strains [51]. Nanoparticle-based systems can co-encapsulate different drugs, ensuring their simultaneous delivery and synergistic action at the infection site. This multi-pronged approach not only enhances bactericidal activity but also reduces the likelihood of resistance development, aligning with the broader goal of combating antimicrobial resistance [9]. Despite these advances, challenges remain in translating nanotechnology-based Trojan horse strategies into clinical practice. Issues such as large-scale manufacturing, biocompatibility, and potential toxicity need to be addressed. Nonetheless, ongoing research underscores the potential of nanocarriers to revolutionize antibiotic delivery by overcoming solubility and stability limitations, protecting drugs from premature degradation, and ensuring targeted delivery [47].

In summary, nanotechnology offers a versatile platform for implementing the Trojan horse strategy in antibiotic therapy. By encapsulating or associating conventional antibiotics within nanocarriers, it is possible to enhance drug stability, solubility, and targeted delivery, thereby overcoming key limitations of traditional formulations. These nanoformulations can traverse biological barriers, deliver drugs directly to infection sites, and even exploit infected cells as delivery vehicles, ultimately improving therapeutic efficacy and mitigating resistance development. As research progresses, these strategies hold promise for transforming infectious disease management and addressing the global challenge of antimicrobial resistance.

## 3. Overcoming Antibiotic Resistance Through Nanotechnology

### 3.1. Overcoming Efflux: Bypassing a Major Resistance Mechanism

The escalating challenge of antimicrobial resistance, particularly mediated by efflux pump mechanisms, necessitates innovative strategies to restore the efficacy of existing antibiotics. Recent advances in nanotechnology have opened promising avenues for bypassing or inhibiting efflux systems, thereby enhancing antimicrobial activity against resistant pathogens. The literature underscored the multifaceted role of nanomaterials in overcoming efflux-mediated resistance, highlighting their potential to revolutionize antimicrobial therapy.

Nanomaterial-based strategies have demonstrated significant promise in circumventing traditional resistance mechanisms, including efflux pumps. According to Parvin et al. [18], nanoparticles can interact with bacterial pathogens in ways that surpass the limitations of conventional antibiotics, notably by overcoming efflux pump mechanisms. These nanostructures can facilitate drug delivery directly into bacterial cells, thereby bypassing the efflux systems that typically expel antibiotics and reduce their intracellular concentrations. Similarly, advanced nanoparticles with multifaceted mechanisms of action have been shown to bypass resistance pathways, including efflux, as discussed by AlQurashi et al. [24]. These mechanisms include disrupting bacterial membranes, generating reactive oxygen species, and delivering antimicrobial agents in a manner that evades efflux recognition.

The role of nanoparticles in overcoming efflux pumps is further supported by studies on polymer-based nanocarriers. For instance, Abo-zeid et al. [52] reported that encapsulating azithromycin into poly (lactic-co-glycolic acid) nanoparticles enhances its antimicrobial activity against efflux-resistant bacteria. The encapsulation process effectively shields the drug from recognition by efflux transporters, allowing higher intracellular concentrations and improved bacterial eradication. This approach exemplifies how nanocarriers can serve as a physical barrier, preventing efflux pump recognition and expulsion of the antimicrobial agent.

Metal and metal oxide nanoparticles have also been extensively investigated for their ability to interact with bacterial cells through mechanisms that bypass efflux systems. Natanzi et al. [53] highlighted that metal nanoparticles, such as silver and zinc oxide, can interact with bacterial membranes and intracellular components, disrupting cellular functions independently of traditional antibiotic pathways. These interactions can lead to increased bacterial cell permeability and damage, reducing reliance on antibiotics that are susceptible to efflux. Moreover, the antimicrobial activity of such nanoparticles is often enhanced when combined with conventional antibiotics, creating synergistic effects that can overcome efflux-mediated resistance.

**Figure 6:**
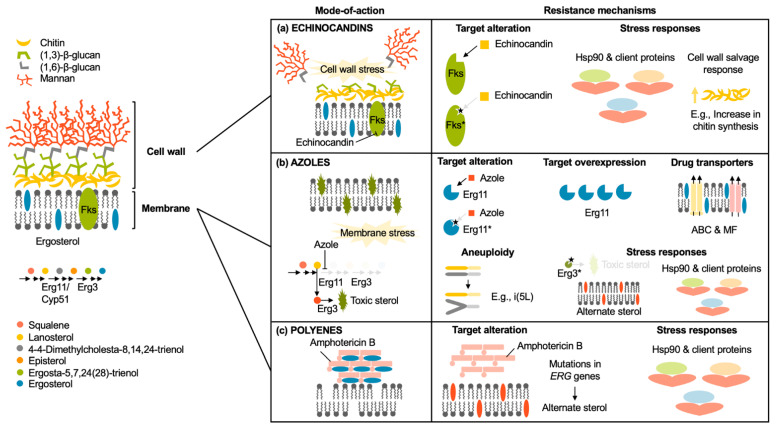
Antifungal mode-of-action and mechanisms of resistance for (**a**) echinocandins, (**b**) azoles, and (**c**) polyenes. Reproduced from [54].

The overexpression of efflux transporters, such as ABC transporters, is a common resistance mechanism in both bacteria and fungi. Lee et al. [54] discussed how nanoparticle formulations can mitigate azole resistance mediated by efflux pumps in fungal pathogens by reducing the reliance on transporter-mediated drug expulsion (Figure 6). Similarly, in bacterial systems, nanoparticles can facilitate drug accumulation within cells, effectively saturating or bypassing efflux systems. This is particularly relevant for multidrug-resistant strains like methicillin-resistant *S. aureus*, where efflux pumps contribute significantly to resistance [55].

Encapsulation of antibiotics into nanocarriers not only enhances drug delivery but also reduces the likelihood of efflux recognition. Abo-zeid et al. [52] and Chakraborty et al. [1] described how nanoparticle encapsulation can shield antibiotics from efflux pumps, thereby increasing their intracellular retention and antimicrobial efficacy. These strategies are especially pertinent for drugs that are substrates for efflux transporters, as nanocarriers can alter pharmacokinetics and cellular uptake pathways, effectively bypassing efflux mechanisms. Furthermore, the integration of nanotechnology with combination therapy approaches offers a promising strategy to combat efflux-mediated resistance. Si et al. [56] emphasized that combining efflux pump inhibitors with nanocarrier-delivered antibiotics can synergistically enhance antimicrobial activity. Nanoparticles can be functionalized with efflux pump inhibitors, which block the expulsion of antibiotics, thereby increasing intracellular drug concentrations and restoring susceptibility.

In summary, the current literature underscored the potential of nanomaterials to overcome efflux pump-mediated resistance through various mechanisms. These include facilitating direct drug delivery into bacterial cells, disrupting membrane integrity, shielding antibiotics from efflux recognition, and enabling synergistic combinations with efflux inhibitors. The multifaceted nature of nanoparticles allows for tailored approaches to bypass or inhibit efflux systems, offering a promising pathway to restore the efficacy of existing antibiotics and combat resistant infections effectively. Continued research into optimizing nanoparticle formulations and understanding their interactions with bacterial resistance mechanisms is essential for translating these strategies into clinical practice.

### 3.2. The Biofilm Challenge: Penetration and Eradication

The challenge of effectively penetrating and eradicating bacterial biofilms remains a significant obstacle in the treatment of persistent infections. Biofilms are complex microbial communities embedded within a self-produced extracellular polymeric substance matrix, which confers protection against antimicrobial agents and host immune responses [57]. This protective environment not only impedes the penetration of conventional antibiotics but also fosters the development of dormant, persistent cells that are highly resistant to treatment [58]. Table 1 presents current therapeutic formulations targeting bacterial biofilms along with their mechanisms of action and efficacy.

One of the primary mechanisms by which biofilms resist antimicrobial agents is through the physical barrier created by the extracellular polymeric substance matrix. This matrix, composed of polysaccharides, proteins, and nucleic acids, acts as a formidable obstacle, restricting the diffusion of antibiotics and other therapeutic agents into the deeper layers of the biofilm [74]. As a result, many antibiotics fail to reach bactericidal concentrations within the biofilm, allowing bacteria, especially dormant cells, to survive and potentially lead to recurrent infections [75].

**Figure 7:**
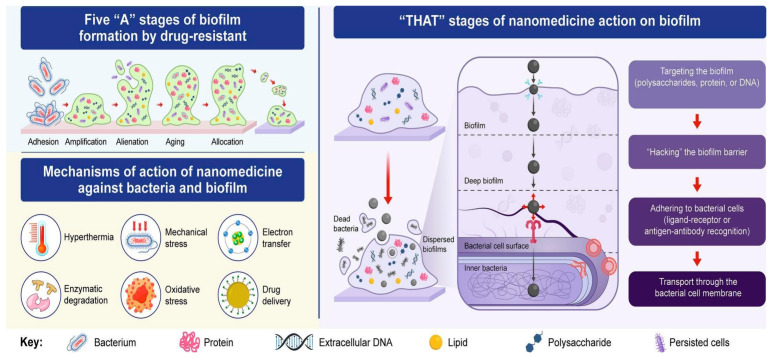
Nanoformulations as disruptors or inhibitors of biofilms. Reproduced from [58].

Nanotechnology offers promising strategies to overcome these barriers (Figure 7). Nanomaterials, particularly nanoparticles, have unique physicochemical properties such as small size, high surface area, and tunable surface functionalities, which enable them to penetrate the extracellular polymeric substance matrix more effectively than traditional antibiotics [76]. For instance, the size of nanoparticles plays a crucial role in their ability to infiltrate biofilms; smaller nanoparticles are generally more capable of navigating through the dense extracellular polymeric substance network [77]. Moreover, surface modifications of nanoparticles, such as the addition of cell-penetrating peptides, further enhance their ability to traverse biofilm barriers and facilitate uptake by bacterial cells [53].

Beyond physical penetration, nanomaterials can be engineered to actively disrupt the biofilm structure. Certain nanoparticles possess intrinsic antimicrobial properties, such as silver, zinc oxide, or titanium dioxide, which can generate reactive oxygen species or release metal ions that damage bacterial cell membranes and intracellular components [78]. These mechanisms not only kill bacteria within the biofilm but also weaken the structural integrity of the extracellular polymeric substance, making subsequent antimicrobial treatments more effective [76]. Targeting dormant and persistent cells within biofilms presents another layer of complexity. These cells are in a phenotypic state that renders them tolerant to many antibiotics, which typically target actively dividing bacteria [58]. Innovative approaches involve designing nanoparticles that can deliver antimicrobial agents directly into these dormant cells or that can induce metabolic activation, rendering them susceptible to conventional antibiotics [58]. For example, some nanocarriers are tailored to release their payloads in response to specific biofilm microenvironment cues, such as pH or enzymatic activity, ensuring localized and controlled drug delivery [79].

Furthermore, combining nanotechnology with other therapeutic strategies enhances biofilm eradication. The use of cell-penetrating peptides attached to nanoparticles facilitates deeper penetration and uptake by bacterial cells within biofilms [53]. Additionally, integrating CRISPR-Cas9 systems with nanocarriers offers a targeted approach to disrupt bacterial gene expression essential for biofilm maintenance and resistance [53]. Despite these advances, challenges remain. The heterogeneity of biofilms, variability in extracellular polymeric substance composition, and potential toxicity of nanomaterials necessitate further research to optimize nanoparticle design for safe and effective clinical application [74]. Moreover, understanding the precise mechanisms by which nanoparticles penetrate and disrupt biofilms is critical for developing next-generation therapeutics. Table 2 presents clinical trials of nanoformulations based anti-biofilm treatments.

In summary, nanomaterials provide a multifaceted approach to overcoming the biofilm barrier. Their ability to physically penetrate the extracellular polymeric substance matrix, actively disrupt biofilm structure, and target dormant cells makes them promising candidates for combating persistent biofilm-associated infections. Continued research into the mechanisms of nanoparticle-biofilm interactions and the development of tailored nanotherapeutics holds the potential to significantly improve treatment outcomes for biofilm-related diseases.

### 3.3. Disarming the Pathogen: Quorum Sensing and Virulence Inhibition

Bacterial infections continue to pose a significant global health challenge, exacerbated by the rise of antimicrobial resistance. A promising alternative therapeutic strategy involves ‘disarming’ pathogens by inhibiting their virulence mechanisms rather than directly killing them, thereby reducing selective pressure for resistance [80]. Central to this approach is the targeting of quorum sensing, a cell density-dependent communication system that bacteria utilize to coordinate collective virulent behaviors [81,82]. By interfering with quorum sensing or quenching its signals, researchers aim to attenuate bacterial pathogenicity and enhance treatment efficacy [81,83]. Recent advancements highlight the potential of antimicrobial nanoformulations in delivering these anti-virulence agents effectively.

Quorum sensing orchestrates various collective behaviors, including the production of virulence factors and biofilm formation [82,83]. In *A. baumannii*, for instance, the quorum sensing process is a key target for novel therapeutic strategies, with enzymes being explored as antimicrobial drugs in various sites within the quorum sensing pathway [81,82]. Similarly, *Burkholderia thailandensis*, a study model for the highly virulent pathogen *B. pseudomallei*, demonstrated that quorum sensing is of prime interest for understanding virulence and developing alternative treatments [84]. The regulation of virulence in *P. aeruginosa*, a pathogen known for its inherent and adapted resistance, is particularly complex. Here, the PqsE enzyme interacts with the quorum-sensing receptor RhlR, and this protein–protein interaction is vital for promoting virulence factor production [85]. Inhibitor mimetic mutations in PqsE, such as PqsE E182W, disrupt this interaction, leading to a failure in virulence factor production [85]. Further studies have determined the molecular basis for this physical interaction, showing how the PqsE-RhlR complex regulates RhlR DNA binding to control virulence factor production [82].

Strategies for quorum sensing inhibition are diverse, encompassing synthetic compounds, natural products, and enzymatic approaches. Quorum quenchers, which hinder the development of virulence or resistance mechanisms without eliminating the microorganisms, are emerging as valuable alternatives to conventional antimicrobials [80]. For Gram-negative pathogens, the preparation of N-acylhomoserine lactone analogs serves as a method for discovering novel quorum quenchers [80]. Small molecules have demonstrated efficacy, with substituted diphenyl amide compounds found to disrupt bacterial quorum sensing and promote survival in a *S. aureus* infection model [86]. Novel β-nitrostyrene derivatives have been designed as potential quorum sensing inhibitors to combat *Serratia marcescens*, effectively inhibiting biofilm formation and downregulating quorum sensing- and biofilm-related genes [87]. Furthermore, chalcone derivatives show promise in attenuating *P. aeruginosa*’s pathogenicity by specifically inhibiting the PqsR component of its quorum sensing system [88]. Antimicrobial peptides have also been shown to fight *P. aeruginosa* at sub-inhibitory concentrations by targeting the anti-quorum sensing pathway, leading to significant inhibition of quorum sensing regulated virulence factors such as pyocyanin, elastase, proteases, and bacterial motility, and inducing structural alterations in the LasR protein [89].

Natural products and medicinal plants are increasingly recognized for their anti-virulence potential [83]. Piperine, for instance, exhibits potent antibiofilm activity against *P. aeruginosa* by accumulating reactive oxygen species, affecting cell surface hydrophobicity, and interfering with quorum sensing [90]. Its antibiofilm activity against *S. aureus* has also been reported [90]. The natural flavonoid phloretin has been shown to inhibit biofilm formation and virulence factors of the cariogenic oral pathogen *S. mutans* [91]. Extracts from *Cinnamomum zeylanicum*, particularly its hexane fraction, can modulate quorum sensing and biofilm formation in Gram-negative bacterial pathogens, providing strong evidence for its potency in targeting bacterial virulence [92]. Similarly, phytochemicals from *Paederia foetida* Linn. demonstrate the ability to inhibit quorum sensing and biofilm formation against multidrug-resistant *A. baumannii* [93].

The integration of these anti-virulence strategies with nanoformulations represents a significant advancement. Nanoformulations can enhance the bioavailability and targeted delivery of quorum sensing inhibitors, improving their efficacy. For example, phytofabricated silver nanoparticles have been shown to significantly attenuate biofilm and quorum sensing regulated virulence factors of the opportunistic pathogen *P. aeruginosa*. At a concentration of 20 µg/mL, silver nanoparticles reported considerable inhibition of biofilm formation, quorum sensing, pyocyanin, and LasB elastase, without impacting the growth of planktonic cells at sub-minimum inhibition concentrations [94]. In another innovative approach, fish gelatin-based nanoformulations, prepared by coating maltol-gold nanoparticles, have been developed. These nanoformulations demonstrated enhanced bioavailability and improved antimicrobial, antibiofilm, and anti-virulence activities. Notably, maltol-gold nanoparticle gel suppressed the expression of genes associated with biofilm formation, quorum sensing, motility, and virulence factors in *P. aeruginosa* at a higher level than maltol-gold nanoparticles alone, corroborating their phenotypic effects [95].

In summary, the strategy of disarming pathogens through quorum sensing and virulence inhibition offers a promising avenue to combat antimicrobial resistance. The diverse array of quorum sensing targets and inhibitory compounds, including synthetic molecules and natural products, underscores the versatility of this approach. Critically, the development of antimicrobial nanoformulations, such as phytofabricated silver nanoparticles and gold nanoparticle-based systems, provides advanced platforms for enhanced delivery and improved efficacy of these anti-virulence agents, paving the way for novel therapeutic interventions against challenging bacterial infections.

### 3.4. Synergistic Interactions: The Multi-Target Advantage

The concept of synergistic interactions in antimicrobial therapy has garnered significant attention, particularly within the realm of nanoformulations that leverage multi-target mechanisms to enhance efficacy. The literature underscored the multifaceted nature of these interactions, highlighting how combining nanomaterials with bioactive compounds or conventional antimicrobials can produce synergistic effects that surpass the capabilities of individual agents.

One prominent theme across the studies is the multi-target approach employed by natural bioactive compounds, especially phenolics and plant-derived molecules. According to research on natural phenolics, these compounds exert their antimicrobial effects through three primary targets: reactive oxygen species generation, membrane disruption, and DNA interaction [96]. The convergence of these mechanisms facilitates a multi-pronged attack on microbial cells, which not only enhances antimicrobial potency but also reduces the likelihood of resistance development. Similarly, plant-based antimicrobials are shown to interact synergistically with metal oxide nanoparticles, amplifying their bactericidal effects [97]. This synergy is attributed to the nanoparticles’ ability to disrupt cell membranes and generate oxidative stress, complementing the bioactive compounds’ multi-target actions.

**Figure 8:**
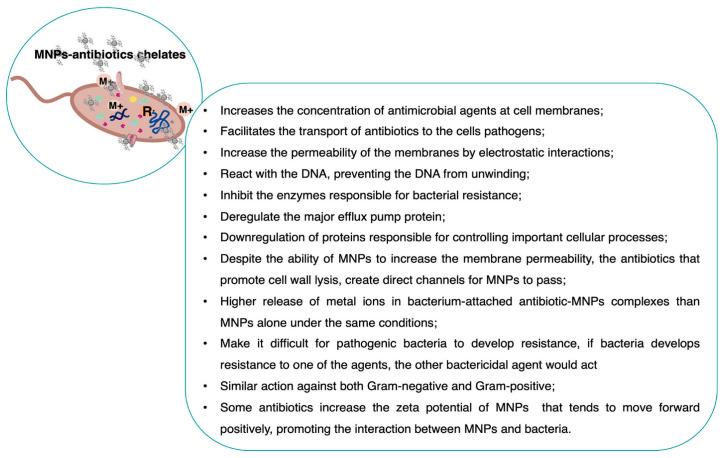
Mechanisms and therapeutic benefits of magnetic nanoparticle and antibiotic conjugates. Reproduced from [99].

Nanotechnology-based strategies further exemplify the multi-target advantage. For instance, daptomycin-loaded nanocarriers demonstrate synergistic killing of methicillin-resistant *S. aureus* by combining membrane targeting with a tailored lipid-based delivery system [98]. This approach enhanced drug delivery efficiency and potentiates antimicrobial activity, illustrating how nanocarriers can serve as platforms for multi-target interactions. Metal nanoparticles, including silver and gold, are frequently highlighted for their ability to synergize with conventional antimicrobial agents. The combination of metal nanoparticles with antibiotics or antifungals can produce multi-layer interactions that enhance microbial eradication and potentially overcome resistance (Figure 8) [99]. These interactions often involve membrane disruption, oxidative stress induction, and interference with microbial DNA or protein synthesis, aligning with the multi-target paradigm. Moreover, the synergistic potential of nanoformulations extends to overcoming antibiotic resistance, as drug combinations targeting multiple bacterial pathways can reduce the likelihood of resistance emergence [100]. Similarly, gold nanoparticles are recognized for their powerful antimicrobial mechanisms and their capacity to synergize with antibiotics, thereby broadening their therapeutic potential [101]. The review emphasized that gold nanoparticles can disrupt bacterial membranes, interfere with intracellular processes, and enhance antibiotic uptake, collectively contributing to a multi-target attack.

Natural compounds such as berberine exemplify the benefits of nanoformulation in achieving multi-target antimicrobial effects. Berberine and its nanoformulations target various bacterial components, including cell walls, DNA, and metabolic pathways, demonstrating a multi-omics basis for their synergistic activity [99]. The nanoformulation enhances bioavailability and facilitates targeted delivery, thereby amplifying the multi-target effects and improving therapeutic outcomes. The integration of phytocomplexes, which contain multiple bioactive molecules, further illustrates the multi-target synergy. Grape seed extracts, for example, exhibit antibacterial profiles that depend on the interactions of individual molecules within the complex, leading to enhanced antimicrobial activity [102]. Understanding these interactions is crucial for designing effective multi-target formulations that exploit the synergistic potential of natural compounds. Recent studies also explored the transcriptomic basis of synergy, revealing that multi-target antibiotic combinations can induce complex interactions at the gene expression level, leading to enhanced bacterial killing [97]. Such insights underscore the importance of understanding molecular mechanisms underlying synergy to optimize nanoformulation strategies.

In summary, the literature highlighted that multi-target interactions are central to the enhanced efficacy of antimicrobial nanoformulations. These interactions involve membrane disruption, oxidative stress, DNA interference, and metabolic pathway inhibition, often facilitated by nanocarriers or combined with natural bioactives and conventional drugs. The synergistic effects not only improve antimicrobial potency but also hold promise for overcoming resistance and reducing drug dosages. As research advances, the design of multifunctional nanoplatforms that harness these multi-target mechanisms is poised to revolutionize antimicrobial therapy, offering more effective and sustainable solutions against resistant pathogens.

## 4. The Immunomodulatory Dimension: Engaging Host Defenses

**Figure 9:**
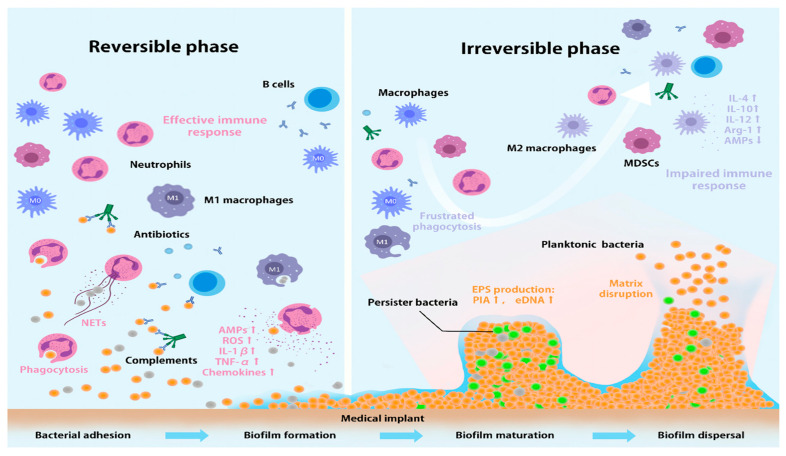
Schematic representation of the host immune response evolves through distinct phases of biofilm formation on implants. Reproduced from [108].

The host immune system plays a critical role in maintaining biological homeostasis by mitigating the detrimental effects of infections [103]. This defense mechanism operates through a coordinated immune response [103], involving an intricate interaction between the innate and adaptive immune systems to enhance the host’s defense capabilities against various pathogens, including certain bacteria [104]. Macrophages, for instance, are vital components of the innate immune system, strategically positioned in tissues, body cavities, and around mucosal surfaces to contribute significantly to host defense [105]. However, challenges arise when the immune system struggles to clear persistent infections, such as biofilm-embedded bacteria [106], or when infections are aggravated by the immune response itself [107]. Moreover, some bacteria are capable of interfering with the host immune response, and even a minor tissue response, particularly in cases like implant-associated infections, can compromise immune defense [108]. Figure 9 presents a schematic representation of how the host immune response evolves through distinct phases of biofilm formation on implants. The progression from initial, reversible bacterial attachment to a stable, irreversible infection correlates directly with the duration of bacterial colonization.

**Figure 10:**
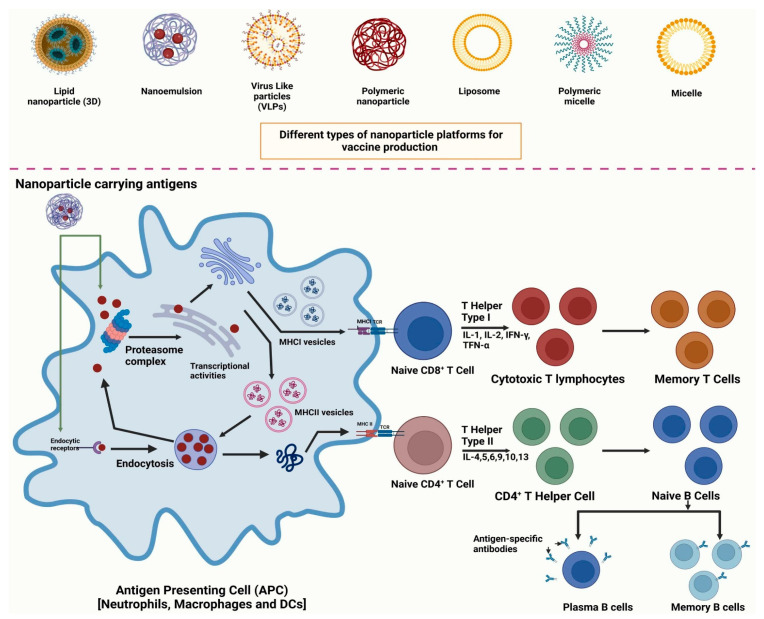
Schematic illustration of the dual activation of innate and adaptive immunity by nanovaccines. Reproduced from [109].

In response to these challenges, immunomodulatory strategies that engage and enhance host defenses are gaining prominence. Host-directed therapies exemplify this approach, offering an effective method for bolstering antiviral defenses and combating viral infections by modifying the host immune response. These therapies aim to boost the host’s natural ability to fight off pathogens [108]. A significant development in this area is the use of nanoformulations to directly stimulate or work in concert with the immune system. Nanovaccines are a prime example, consisting of nanoparticles engineered to associate with or encapsulate components that stimulate the host’s immune system (Figure 10) [109]. This directly highlighted how nanoformulations can activate the immune response, thereby contributing to the clearance of microbial infections [109]. The deliberate design of these nanoparticles allows for targeted delivery and enhanced immune cell engagement, leading to a more robust and effective immune response.

Beyond synthetic nanoformulations, naturally occurring nanoscale entities like bacteriophages also demonstrate significant immunomodulatory potential. Phage therapy is explored as a strategy for managing infections where the host’s immune system proves inadequate, such as in clearing biofilm-embedded bacteria [106]. Bacteriophages are capable of modulating host immune responses, which in turn enhances bacterial clearance [106]. Specifically, bacteriophages can act synergistically with the innate immune response to promote the clearance of bacterial infections [110]. During phage therapy, the activity of neutrophils, a type of white blood cell, is observed to contribute to the clearance of bacterial infections [110]. This synergy between bacteriophages and innate immune cells underscores how nanoscale biological agents can work in concert with the host’s immune system to overcome microbial threats.

It is also understood that the efficacy of the immune system is closely linked to the host’s overall health, with proper immune function being dependent on good nutritional status [107]. While the host immune system is a sophisticated defense mechanism, its interaction with pathogens is complex. For example, lipopolysaccharides, which are large amphipathic glycoconjugates, form important outer membrane components of Gram-negative bacteria and are potent immune stimulators [111]. Understanding these interactions is crucial for developing effective immunomodulatory strategies.

In summary, the immunomodulatory dimension offers promising avenues for engaging host defenses to clear microbial infections. Strategies involving nanoformulations, such as nanovaccines, directly stimulate the host immune system. Furthermore, nanoscale biological agents like bacteriophages modulate host immune responses and work synergistically with innate defenses to enhance bacterial clearance. By harnessing and enhancing these intrinsic host defense mechanisms, researchers and clinicians aim to overcome the challenges posed by persistent and resistant microbial infections, ultimately promoting optimal host health. This integrated approach, focusing on empowering the host’s own immune system, represents a significant paradigm shift in infectious disease management.

## 5. Navigating the Immune Hurdle: Opsonization, Clearance, and Stealth Strategies

Navigating the immune hurdles associated with antimicrobial nanoformulations necessitates a comprehensive understanding of mechanisms such as opsonization, immune clearance, and the development of stealth strategies. Recent literature underscores the critical role of surface engineering and physicochemical modifications in modulating nanoparticle interactions with the immune system, thereby enhancing their therapeutic efficacy.

**Figure 11:**
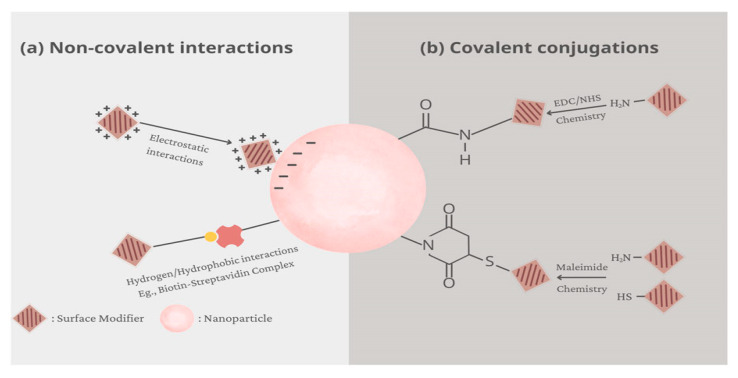
Schematic illustration of 2 surface modification strategies: (**a**) electrostatic adsorption and (**b**) covalent conjugation. Reproduced from [113].

One of the primary challenges faced by nanoformulations is opsonization, a process where serum proteins bind to the surface of nanoparticles, marking them for recognition and clearance by immune cells. As highlighted by Mehta et al. [49], opsonization is a significant barrier in drug and gene delivery systems, often leading to rapid clearance from circulation. The formation of a protein corona not only influences the biodistribution of nanoparticles but also determines their recognition by phagocytic cells. To mitigate this, surface modification strategies such as PEGylation have been extensively employed. For instance, Huq et al. [112] discusses how PEGylation of mesoporous silica nanoparticles reduces opsonization, thereby prolonging circulation time and enhancing delivery efficiency. Similarly, surface decoration techniques (Figure 11), as elaborated by Ly et al. [113], focus on functionalizing nanoparticle surfaces to improve physicochemical stability and biological interactions, ultimately reducing immune recognition.

Surface properties are pivotal in dictating how nanoparticles interact with the immune system. The surface engineering of nanoparticles influences their recognition by immune cells, interaction with target tissues, and response under pathological conditions [114]. Modulating surface charge, hydrophobicity, and functional groups can either promote immune evasion or facilitate targeted immune responses, depending on therapeutic goals. For example, lipid-based nanoparticles, such as nanoliposomes, have been tailored with stealth layers to evade immune detection. As noted by Sowmiya et al. [115], nanoliposomes can be engineered to enhance stability and reduce opsonization, thereby improving their capacity to deliver antimicrobial agents effectively.

Beyond surface modifications, the development of stealth strategies is crucial for navigating immune clearance. The concept of ‘stealth’ layers, such as polyethylene glycol coatings, is designed to minimize protein adsorption and immune cell recognition [116]. Polymer-based nanoparticles, including those made from biocompatible polymers, can be further functionalized with targeting ligands or protective coatings to enhance their immune evasion capabilities. These modifications not only extend circulation time but also improve the accumulation of nanoparticles at infection sites, leveraging the enhanced permeability and retention effect, which is particularly advantageous in tumor and infection microenvironments [117].

Advancements in nanotechnology have also focused on utilizing natural and plant-derived compounds to enhance antimicrobial efficacy while circumventing immune clearance. As discussed by Parvin et al. [118], phytochemical-loaded nanoparticles combine intrinsic antimicrobial properties with the ability to modulate immune responses, potentially reducing the likelihood of immune-mediated clearance. Such strategies exemplify the integration of biological and nanotechnological approaches to develop multifaceted mechanisms that can both evade immune detection and exert potent antimicrobial effects.

Furthermore, the immune microenvironment plays a significant role in determining the fate of nanoformulations. The immune response can be modulated through surface engineering to either promote immune activation against pathogens or suppress unwanted immune reactions that lead to clearance [119]. For example, in the context of central nervous system applications, recent advancements have demonstrated how nanoparticles can be designed to navigate immune barriers effectively, balancing immune modulation with therapeutic delivery [119].

In summary, the current literature emphasizes that overcoming immune hurdles in antimicrobial nanoformulations hinges on sophisticated surface engineering, strategic use of stealth coatings, and leveraging biological insights into immune recognition mechanisms. Techniques such as PEGylation, surface decoration, and the incorporation of phytochemicals are central to reducing opsonization and immune clearance. Simultaneously, designing nanoparticles with tailored surface properties can facilitate targeted delivery and immune evasion, thereby enhancing therapeutic outcomes. As nanotechnology continues to evolve, integrating these strategies will be vital for developing multifaceted antimicrobial agents capable of navigating the complex immune landscape effectively.

## 6. The Resistance Counter-Offensive: Microbial Adaptation to Nanomaterials

The ongoing battle between microbial pathogens and antimicrobial agents has become increasingly complex with the advent of nanomaterials, which are being explored as innovative antibacterial strategies. However, bacteria have demonstrated remarkable adaptability, developing resistance mechanisms that counteract the efficacy of nanomaterials. The literature review presents current understanding of microbial resistance to nanomaterials, focusing on three primary mechanisms: biofilm matrix alteration, surface mutation, and efflux of dissolved ions.

Biofilm formation is a central factor in microbial resistance, significantly contributing to the persistence of infections and the ability of bacteria to withstand antimicrobial interventions. According to Uruén et al. [120], biofilms are structured communities of microorganisms embedded within a self-produced extracellular polymeric substance matrix. This matrix, composed mainly of polysaccharides, proteins, and nucleic acids, creates a protective environment that imparts tolerance against antibiotics, disinfectants, and immune responses [121]. The structural complexity of biofilms allows bacteria to modify their immediate environment, effectively acting as a physical and chemical barrier. Such modifications can impede the penetration of nanomaterials, reducing their bactericidal effectiveness. For instance, the extracellular polymeric substance matrix can adsorb or neutralize reactive nanomaterials, diminishing their capacity to interact with bacterial cells [122].

Furthermore, bacteria within biofilms can undergo specific alterations in their matrix composition, which enhances resistance. The production of polysaccharides and other matrix components can be upregulated in response to nanomaterial exposure, reinforcing the biofilm’s protective barrier [122]. This adaptive response exemplifies how biofilm matrix alteration serves as a resistance mechanism, enabling bacteria to survive in hostile environments created by nanomaterials designed to disrupt cellular functions.

Surface mutation represents another critical resistance strategy. Bacteria can acquire genetic changes that modify cell surface structures, thereby reducing nanomaterial binding or uptake. Such mutations can alter membrane proteins, lipopolysaccharides, or other surface molecules, decreasing the affinity of nanomaterials for bacterial cells [10]. These surface modifications can prevent nanomaterials from effectively attaching or penetrating bacterial membranes, which is essential for their antimicrobial activity. The dynamic nature of bacterial genomes allows rapid adaptation through mutations, especially under selective pressure exerted by nanomaterials [10].

In addition to structural modifications, bacteria can actively expel toxic substances, including dissolved ions released from nanomaterials, through efflux mechanisms. Efflux pumps are membrane proteins that transport a variety of compounds out of the cell, thereby reducing intracellular concentrations of potentially harmful agents [10]. When nanomaterials release ions such as silver or copper, bacteria can respond by upregulating efflux systems, effectively decreasing the intracellular accumulation of these ions and mitigating their antimicrobial effects. This ion efflux mechanism is a sophisticated form of resistance, allowing bacteria to survive in environments with high concentrations of nanomaterial-derived ions [10].

A common critique of the antimicrobial nanoformulations is the assumption that multi-target mechanisms inherently preclude the development of resistance. While the simultaneous disruption of multiple bacterial structures—such as membranes, DNA, and metabolic enzymes—certainly raises the evolutionary barrier, emerging evidence suggests that bacteria are not defenseless against even these multifaceted agents. Over prolonged or repeated exposure, bacterial populations can evolve adaptive strategies that circumvent the very complexity of nanoformulations.

One pathway to resistance involves the coordinated upregulation of general stress response systems. Exposure to sub-lethal concentrations of nanoparticles has been shown to activate SoxS and RpoS regulons, which orchestrate defenses against oxidative stress, membrane damage, and protein misfolding [123]. Over time, constitutive activation of such pathways can confer tolerance not only to the original nanomaterial but also to structurally unrelated agents, a phenomenon akin to adaptive resistance. Furthermore, bacteria can reduce the internalization of nanoparticles by modifying membrane fluidity or capsule composition, thereby diminishing the intracellular concentration of reactive species or dissolved ions before they can engage multiple targets [124].

Another concerning development is the horizontal transfer of resistance determinants. Sub-lethal exposure to metal nanoparticles has been implicated in the co-selection of antibiotic resistance genes via mobile genetic elements. For instance, silver and copper nanoparticles can induce oxidative stress that upregulates efflux pumps and integrons, facilitating the spread of resistance cassettes across bacterial populations [125,126]. This suggests that the selective pressure exerted by nanomaterials may inadvertently promote the dissemination of multidrug resistance, undermining their long-term utility.

Moreover, the multi-target nature of some nanoformulations may be partially neutralized by bacterial ‘tolerance’ mechanisms that do not directly counteract the nanomaterial but instead reduce its effective impact. For example, bacteria entering a persister state within biofilms can survive exposure to otherwise lethal concentrations of nanoparticles by slowing their metabolic activity, thereby limiting the uptake and intracellular activity of the nano-agent [120,121]. While this does not represent genetic resistance per se, it enables survival under therapeutic challenge and provides an evolutionary reservoir from which true resistance can emerge.

The interaction between nanomaterials and bacterial resistance mechanisms is further complicated by environmental factors. For example, nanomaterials can undergo physical and chemical transformations, such as photo-oxidation, which may influence their antimicrobial activity and the bacterial response. The adsorption of environmental contaminants onto nanoplastics and nanomaterials can also mediate transport and potentially impact resistance development [127].

Recent advances in nanotechnology have aimed to overcome bacterial resistance by designing nanomaterials with enhanced properties. For instance, gallium-based nanomaterials have been shown to disrupt biofilm matrices and interfere with bacterial metabolic processes, presenting a multi-pronged approach to combat resistance [122]. Combining nanomaterials with traditional antibiotics can also lower the required doses and reduce the likelihood of resistance development [123]. Nonetheless, bacteria’s ability to adapt through biofilm matrix modification, surface mutation, and efflux of ions underscores the need for ongoing research into resistance mechanisms.

In summary, microbial resistance to nanomaterials involves complex, multifaceted strategies primarily centered around biofilm matrix alteration, surface mutations, and efflux of dissolved ions. These mechanisms enable bacteria to survive in environments laden with nanomaterials, posing significant challenges to their effective application. Understanding these adaptive responses is crucial for developing next-generation nanomaterials capable of circumventing bacterial defenses and ensuring sustainable antimicrobial efficacy.

## 7. The Nano-Bio Interface: The Defining Role of Surface Physicochemistry

The nano-bio interface is fundamentally governed by the physicochemical characteristics of nanoparticles, notably size, shape, surface charge, and surface functionalization. These parameters critically influence the initial interaction with microbial cells, determine the mechanism of action, and ultimately dictate the efficacy of nanoparticle-based interventions. An understanding of these factors is essential for optimizing nanoparticle design for antimicrobial and therapeutic applications.

### 7.1. Size of Nanoparticles and Its Biological Implications

Size is a pivotal determinant in nanoparticle interactions with biological systems, including microbial cells. Smaller nanoparticles tend to have a higher surface area-to-volume ratio, which enhances their reactivity and interaction potential with microbial membranes. According to Dolai et al. [128], nanoparticle size influences their interaction with biological interfaces through surface chemical groups, with smaller particles exhibiting increased surface reactivity. This heightened reactivity facilitates more effective engagement with microbial cell envelopes, potentially leading to enhanced antimicrobial activity. Similarly, pharmacokinetic studies highlighted by Haripriyaa et al. [129] emphasized that an optimal particle size can improve drug entrapment efficacy and influence biodistribution, which is crucial for therapeutic applications. The ability of nanoparticles to penetrate biological barriers, such as the blood–brain barrier or microbial cell walls, is also size-dependent, with smaller particles generally demonstrating superior permeability [130].

### 7.2. Shape as a Modulator of Interaction and Mechanism

The shape of nanoparticles influences how they interact with microbial cells and their subsequent mechanism of action. For instance, rod-shaped or elongated nanoparticles may exhibit different adhesion and penetration behaviors compared to spherical counterparts. As noted by Chehelgerdi et al. [131], the shape of nanocarriers affects their targeting efficiency and drug release profiles. The surface interactions are also shape-dependent, with certain geometries facilitating more effective contact with microbial membranes, thereby enhancing antimicrobial efficacy. Ray et al. [132] underscored that surface reactivity and shape collectively influence nanoparticle interactions with biological surfaces, including microbial cell envelopes, which can modulate the mechanism of microbial disruption or internalization.

### 7.3. Surface Charge and Its Role in Biointeractions

Surface charge is a critical factor influencing nanoparticle affinity for microbial cells, which often possess negatively charged cell envelopes. Positively charged nanoparticles tend to exhibit stronger electrostatic interactions with microbial membranes, leading to increased membrane disruption or internalization. As discussed by Chaudhary et al. [133], silver nanoparticles exert antimicrobial effects partly through surface interactions that facilitate the release of silver ions and direct contact with pathogens. The surface charge also affects the stability and aggregation behavior of nanoparticles in biological environments, impacting their bioavailability and activity [134]. Furthermore, the surface charge modulates immune recognition and clearance, which can influence the overall antimicrobial efficacy [132].

### 7.4. Surface Functionalization and Its Multifaceted Impact

Surface functionalization involves the modification of nanoparticle surfaces with specific chemical groups or biomolecules to tailor their interactions with microbial cells. This customization can enhance targeting specificity, improve stability, and modulate the mechanism of action. For example, Mitchell et al. [135] highlighted that functionalization strategies enable nanoparticles to navigate complex biological barriers and improve drug delivery precision. In antimicrobial contexts, surface modifications can facilitate the attachment of antimicrobial agents or ligands that target microbial surface structures, thereby increasing efficacy [1]. Additionally, functionalization can influence the nanoparticle’s surface charge and hydrophobicity, further affecting biointeractions and cellular uptake [136].

### 7.5. Interplay of Size, Shape, Surface Charge, and Functionalization

The combined effects of these parameters create a complex landscape that determines the nano-bio interface. For instance, the interaction of silver nanoparticles with *E. coli* involves considerations of size, shape, surface charge, and surface chemistry, which collectively influence their ability to disrupt cell envelopes and exert antimicrobial effects [1]. Similarly, the design of mesoporous silica nanoparticles leverages tunable synthesis parameters to optimize surface area, functionalization, and particle morphology for targeted applications [136].

In summary, the initial interaction of nanoparticles with microbial cells is intricately dictated by their size, shape, surface charge, and surface functionalization. These parameters influence not only the physical contact and adhesion but also the subsequent mechanisms—such as membrane disruption, internalization, or ion release—that underpin antimicrobial and therapeutic efficacy. Advances in nanotechnology continue to refine our understanding of these relationships, enabling the rational design of nanoparticles that can effectively navigate biological barriers, target pathogens, and deliver therapeutic payloads with high precision. The synergy of these physicochemical properties underscores the importance of a holistic approach to nanoparticle engineering for biomedical applications, particularly in combating microbial resistance and enhancing drug delivery systems.

## 8. Beyond Bacteria: Mechanistic Action Against Fungi, Viruses, and Parasites

**Figure 12:**
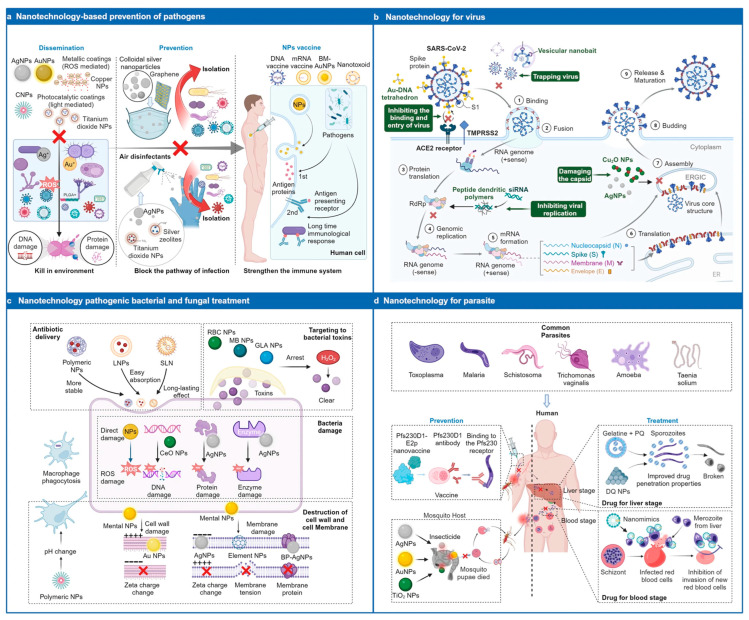
Antimicrobial nanoformulations: applications against bacteria, fungi, viruses, and parasites. Reproduced from [17].

The exploration of nanoformulations as therapeutic agents extends beyond their well-documented efficacy against bacteria, encompassing a broad spectrum of pathogenic classes such as fungi, viruses, and parasites (Figure 12). Recent literature underscores the multifaceted mechanisms through which nanomaterials exert antimicrobial effects, highlighting their potential to address the escalating challenge of antimicrobial resistance across diverse microbial domains.

While primarily studied against bacteria, similar membrane-targeting actions have been observed against fungi and parasites, suggesting a broad-spectrum potential. Gold nanoparticles, beyond their aesthetic appeal, have been identified as potent agents against fungi and parasites, with synergistic interactions documented against viral and fungal pathogens [101]. These interactions often involve the physical disruption of pathogen membranes or interference with membrane-associated processes, which are conserved across various microbial classes.

In addition to membrane disruption, nanomaterials can induce oxidative stress within pathogens. Zinc oxide nanoparticles, for example, exert their antimicrobial effects partly through reactive oxygen species generation, which damages cellular components such as lipids, proteins, and nucleic acids [28]. This oxidative mechanism is not limited to bacteria but extends to fungi and parasites, where reactive oxygen species mediated damage compromises pathogen viability.

Another significant mechanism involves the interaction of nanomaterials with microbial genetic material. Certain nanoparticles can penetrate microbial cells and interfere with DNA or RNA synthesis, thereby inhibiting replication and transcription processes. Although specific details on this mechanism against fungi, viruses, and parasites are limited in the provided documents, the general principle aligns with the known capacity of nanomaterials to disrupt intracellular functions across different pathogen types [101].

Antimicrobial peptides, naturally occurring molecules with broad-spectrum activity, exemplify mechanisms that can be mimicked or enhanced by nanoformulations. Antimicrobial peptides act through multiple mechanisms, including membrane permeabilization, intracellular targeting, and immune modulation [26,137,138]. Their ability to target fungi, parasites, and viruses is well-documented, with mechanisms such as pore formation and interference with vital intracellular processes. Nanoparticles can serve as delivery vehicles for antimicrobial peptides or be engineered to mimic their activity, thereby broadening their antimicrobial spectrum beyond bacteria [26,137,138].

The immune system’s innate and adaptive responses also play a crucial role in combating non-bacterial pathogens. Complement activation and opsonization facilitate pathogen clearance, and nanomaterials can augment these natural defenses. For example, nanostructures can enhance immune recognition or deliver immunomodulatory agents to infected tissues, thereby strengthening host defenses against fungi, viruses, and parasites [17,104]. Furthermore, the development of nanomaterials with specific targeting capabilities enhances their efficacy against diverse pathogens. Surface modifications enable selective binding to pathogen-specific markers, facilitating targeted delivery of antimicrobial agents or disrupting pathogen-specific structures. Such strategies are particularly promising against fungi and parasites, which often possess unique cell wall components or surface antigens [101].

In summary, the mechanisms employed by nanoformulations against fungi, viruses, and parasites are multifaceted and often synergistic. They include physical disruption of membranes, induction of oxidative stress, interference with genetic material, and immune system modulation. The versatility of these mechanisms, coupled with advances in nanotechnology, positions nanoformulations as promising candidates for broad-spectrum antimicrobial therapies. Continued research into these mechanisms will be essential to optimize their efficacy and minimize potential toxicity, ultimately contributing to the global effort to combat antimicrobial resistance across all pathogenic classes.

## 9. Computational and In Silico Models: Predicting Mechanisms and Optimizing Design

The advent of computational and in silico modeling has revolutionized the development of antimicrobial nanoformulations, providing powerful tools for understanding mechanisms of action and optimizing nanoparticle design. These approaches leverage advanced algorithms, molecular simulations, and machine learning techniques to predict physicochemical properties, biological interactions, and efficacy, thereby accelerating the development process and reducing reliance on extensive experimental trials.

One of the primary applications of in silico strategies in nano-drug design involves predicting the mechanisms by which nanomaterials exert antimicrobial effects. Molecular modeling, in particular, encompasses a suite of computational methodologies aimed at elucidating the structural interactions between nanoparticles and microbial targets [139]. For instance, molecular dynamics simulations have been employed to understand how nanoparticles interact with bacterial membranes, revealing insights into membrane disruption and nanoparticle penetration pathways. Such models facilitate the prediction of antimicrobial mechanisms, including oxidative stress induction, membrane destabilization, and ion release, which are critical for designing effective nanoantimicrobials [139].

In addition to mechanistic insights, computational models are instrumental in predicting the physicochemical properties of nanoparticles, such as size, shape, surface charge, and stability, which directly influence their antimicrobial activity. Artificial intelligence algorithms, including machine learning models, have been utilized to predict these properties based on synthesis parameters, enabling the rational design of nanoparticles with desired characteristics [140]. These models help identify optimal synthesis conditions and predict how modifications in nanoparticle composition or surface functionalization can enhance antimicrobial efficacy.

The synthesis and optimization of specific nanomaterials, such as silver nanoparticles, have benefited from computational modeling approaches. For example, models predicting the antibacterial potential of silver nanoparticles have been developed to understand how factors like particle size and surface chemistry influence their activity [141]. Such models not only elucidate potential mechanisms—such as reactive oxygen species generation and membrane interaction—but also guide the synthesis process to produce nanoparticles with enhanced antimicrobial properties. This integration of modeling with experimental synthesis exemplifies how computational tools can streamline the development of effective antimicrobial nanomaterials.

Artificial intelligence and machine learning have emerged as pivotal in predicting and optimizing nanoparticles based antimicrobial agents. Artificial intelligence driven models can analyze large datasets to identify patterns correlating nanoparticle features with antimicrobial activity, thus enabling the design of more potent formulations [142,143]. These models can also predict toxicity and biocompatibility, which are crucial for clinical translation. For instance, AI algorithms have been applied to optimize nanoparticle size and surface modifications to maximize microbial targeting while minimizing adverse effects [143].

Furthermore, in silico methods extend to the simulation of nanoparticle interactions within complex biological environments. Multi-scale modeling approaches integrate molecular-level interactions with larger-scale pharmacokinetic and pharmacodynamic simulations, providing comprehensive insights into nanoparticle behavior in vivo [144]. Such models are particularly valuable in designing drug delivery systems that can effectively target infections while minimizing off-target effects. They enable researchers to simulate how nanoparticles traverse biological barriers, release antimicrobial agents, and interact with microbial communities, thus informing the rational design of more effective formulations [144].

The role of computational modeling in the synthesis and preparation of antimicrobial nanomaterials is also noteworthy. For example, models predicting the optimal synthesis parameters for silver nanoparticles have been used to enhance their antimicrobial efficacy [141]. Additionally, molecular dynamics simulations help predict the compatibility and distribution of nanoparticles within polymer matrices, which is essential for developing composite materials with sustained antimicrobial activity [145]. These models facilitate the rational design of nanocomposites and coatings, ensuring uniform distribution and stability of antimicrobial agents.

Recent advancements have also integrated bioinformatics and artificial intelligence to improve the design of antimicrobial agents beyond traditional nanomaterials. For instance, computational biology approaches have been employed to predict microbial resistance mechanisms and guide the development of nanoparticles capable of overcoming resistance [146]. Similarly, metabolic and genomic modeling combined with machine learning have been used to optimize microbial interactions with nanomaterials, leading to more effective antimicrobial strategies [147].

In summary, the integration of computational and in silico models has significantly advanced the field of antimicrobial nanoformulations. These models provide mechanistic insights, predict physicochemical and biological properties, and guide the rational design and synthesis of nanoparticles with enhanced antimicrobial activity. The synergy between molecular modeling, artificial intelligence, and multi-scale simulations offers a promising pathway toward developing next-generation antimicrobial nanomaterials that are both effective and safe. As computational methodologies continue to evolve, their role in accelerating the discovery and optimization of nanoantimicrobials is poised to expand further, ultimately contributing to more effective strategies against resistant microbial pathogens.

## 10. The Translational Pathway: From Mechanistic Promise to Clinical Reality

The development and application of antimicrobial nanoformulations hold significant promise for addressing the escalating challenge of antibiotic resistance and enhancing targeted drug delivery. However, translating these nanotechnologies from laboratory research to clinical practice involves navigating a complex landscape of safety concerns, scalability issues, and regulatory hurdles. The current literature underscores that while nanomaterials offer innovative solutions, their practical implementation is impeded by multifaceted challenges that must be systematically addressed.

One of the primary concerns in the translational pathway of antimicrobial nanoformulations is nanotoxicology. The unique physicochemical properties of nanoparticles, such as size, shape, surface charge, and composition, influence their biological interactions and potential toxicity. As highlighted Parvin et al. [18], concerns related to toxicity are a significant barrier, with nanomaterials needing thorough evaluation to ensure safety before clinical application. Similarly, Desai [148] emphasizes that toxicity concerns are central to the development of nanoparticle-based therapeutics, as adverse biological responses could compromise patient safety and hinder regulatory approval. The review by Havelikar et al. [149] further elaborates on the complexities of nanotoxicity, exploring mechanisms of toxicity and associated ethical issues, which are critical for establishing safety profiles. These studies collectively indicate that understanding and mitigating nanotoxicity is essential for the successful translation of antimicrobial nanoformulations.

**Figure 13:**
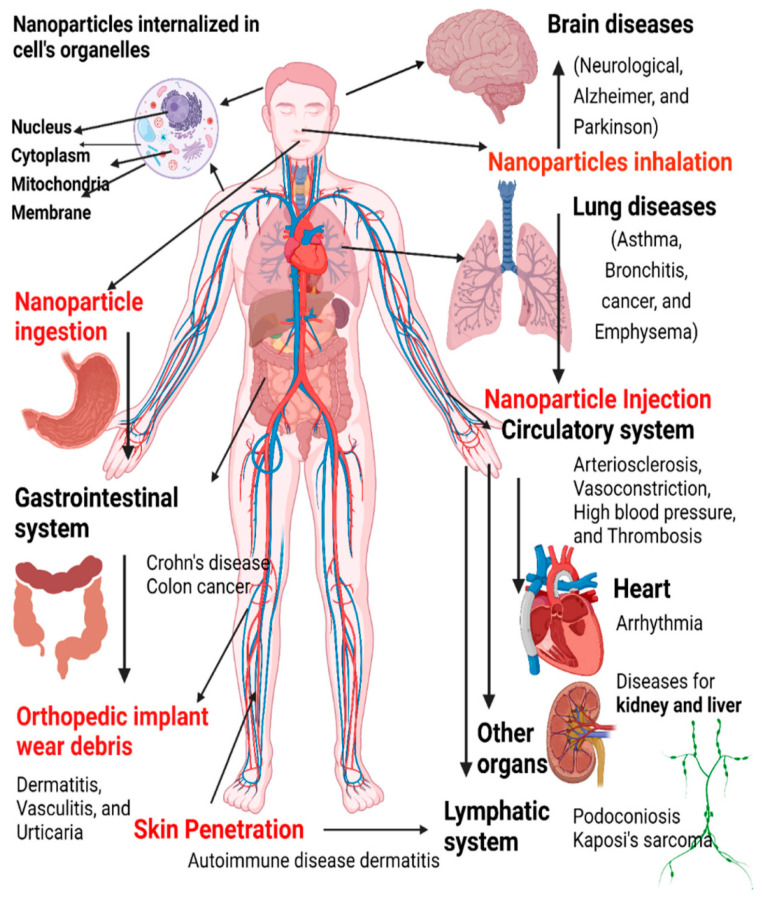
Potential adverse health effects from nanoparticle exposure can occur via inhalation, dermal contact, or ingestion, leading to a spectrum of biological hazards. Reproduced from [153].

Safety assessment of nanomaterials involves evaluating their interactions with biological systems, including potential for bioaccumulation, off-target effects, and environmental impact. For instance, Januja et al. [130] discusses silica nanoparticles and their ability to traverse biological barriers such as the blood–brain barrier and skin, raising concerns about unintended tissue accumulation and toxicity. The review underscores the importance of tailoring nanoparticle properties to minimize adverse effects while maintaining therapeutic efficacy. Moreover, Laib et al. [150] highlights that silver nanoparticles, despite their broad antimicrobial potential, face safety challenges that must be addressed through careful design and testing to prevent toxicity-related setbacks (Figure 13).

Scalability remains another critical challenge in bringing antimicrobial nanoformulations to widespread clinical use. Producing nanomaterials with consistent quality, size distribution, and surface characteristics at an industrial scale is complex and costly. As noted by Laib et al. [150], large-scale production of silver nanoparticles requires overcoming technical hurdles to ensure uniformity and reproducibility, which are vital for regulatory approval and clinical reliability. Elumalai et al. [151] discusses strategies to enhance nanoparticle stability, which is also pertinent to scalability, as stable formulations are easier to manufacture, store, and distribute. Achieving scalable manufacturing processes that preserve nanoparticle functionality without compromising safety is thus a key obstacle that must be addressed through innovative engineering and process optimization.

Regulatory hurdles constitute a significant bottleneck in the clinical translation of nanomedicines. Unlike conventional drugs, nanomaterials often fall into a regulatory gray area due to their complex structures and interactions with biological systems. Rodríguez-Gómez et al. [152] provides insights into existing regulatory pathways and guidelines, emphasizing that current frameworks are still evolving to accommodate nanotechnology-based therapeutics. The lack of standardized testing protocols and clear regulatory criteria complicates approval processes, as highlighted by Parvin et al. [18] and Desai [148]. Furthermore, Desai et al. [55] advocates for a ‘one health’ approach, integrating safety, environmental impact, and regulatory considerations to streamline the pathway for antimicrobial nanomedicines. The need for comprehensive, harmonized regulatory standards is critical to facilitate safe and efficient translation from bench to bedside.

In addition to safety and regulatory issues, the literature points to the importance of designing nanoparticles with enhanced stability and targeted delivery capabilities. Liu et al. [154] discusses advances in nanoparticle-based drug delivery systems, emphasizing that precise targeting can reduce off-target effects and toxicity. Similarly, Elumalai et al. [151] explores methods to improve nanoparticle stability, such as protective coatings, which are essential for maintaining efficacy during manufacturing, storage, and in vivo application. These strategies are vital for overcoming practical challenges related to formulation stability and ensuring consistent therapeutic outcomes.

In summary, the translational pathway for antimicrobial nanoformulations is fraught with practical challenges that encompass nanotoxicology, scalability, and regulatory hurdles. Addressing nanotoxicity requires comprehensive safety assessments and innovative design to minimize adverse effects. Scalability demands the development of reproducible manufacturing processes capable of producing high-quality nanomaterials at an industrial scale. Regulatory pathways need to be clarified and harmonized to accommodate the unique features of nanomedicines, facilitating their approval and clinical adoption. The literature collectively underscores that overcoming these challenges necessitates a multidisciplinary approach, integrating advances in nanotechnology, toxicology, manufacturing, and regulatory science to realize the full potential of antimicrobial nanoformulations in healthcare.

## 11. The Ecological Impact: Fate, Transport, and Environmental Nanotoxicology of Nano-Antimicrobials

The ecological impact of nano-antimicrobials encompasses their complex fate, transport mechanisms, and potential environmental toxicity, which are critical for understanding their environmental nanotoxicology. Recent literature provided insights into how these nanomaterials interact with various environmental media, their pathways of movement, and the associated ecological risks.

### 11.1. Environmental Release: Pathways into Ecosystems

Nano-antimicrobials enter environmental systems through multiple interconnected pathways that span the entire lifecycle of these materials, from manufacturing through consumer use to final disposal. The primary conduit for environmental release is the wastewater treatment infrastructure, which serves as both a processing points and a distribution hub for these engineered nanomaterials [155]. Most engineered silver nanoparticles used in consumer products, medical applications, and industrial processes ultimately find their way to wastewater treatment plants, where they become concentrated in sewage sludge. This sludge then acts as the main vector delivering engineered silver to agricultural soils when biosolids are applied as fertilizer, creating a pathway from urban centers to rural farmland. Notably, the mass of engineered silver nanoparticles in soils is typically about three orders of magnitude lower than the total silver content in sludge-amended soils, reflecting significant transformation processes that occur during wastewater treatment [155].

The journey of nano-antimicrobials through wastewater treatment facilities involves substantial chemical transformation that fundamentally alters their environmental behavior. A large fraction of engineered silver nanoparticles undergoes sulfidation to form silver sulfide during treatment processes, a transformation that markedly reduces the release of soluble silver ions [155]. This sulfidation process is critical because it substantially decreases the acute bioavailability of silver, with measured soluble silver concentrations in sludge-treated soils generally remaining at or below 6 μg/L in reviewed studies. However, this transformation does not eliminate environmental risk entirely; rather, it shifts the nature of the exposure from highly bioavailable ionic silver to more persistent particulate forms that may pose different long-term risks [155,156].

Beyond wastewater pathways, direct release from consumer and medical products represents another significant route of environmental entry. Textiles, coatings, personal care items, and medical materials containing nano-antimicrobials continuously shed nanoparticles into washing and cleaning effluents or during disposal [157,158]. Silver-containing materials have been documented to perturb microbial communities in activated sludge, consistent with significant partitioning of silver nanoparticles to sludge and subsequent downstream environmental exposure via biosolid application or effluent discharge [157,158]. However, a critical gap exists in our understanding of release from medical sources specifically. Current literature does not provide robust, reproducible measurements of nanoparticle mass released per patient through excretion or per disposed medical device across representative healthcare settings. This absence of quantitative data on patient excretion and medical device disposal represents a significant limitation in our ability to model total environmental burden and to develop targeted mitigation strategies for healthcare-associated releases.

The manufacturing sector also contributes to environmental loading, though again, specific release rates from production facilities are inconsistently reported across the literature. Laboratory and full-scale wastewater treatment plant experiments consistently demonstrate that silver-containing materials can significantly alter microbial community structure and function in treatment systems, providing indirect evidence of substantial release quantities [157,158]. These perturbations to wastewater treatment microbiology raise concerns not only about direct environmental impacts but also about potential effects on treatment efficiency and the quality of treated effluent entering receiving waters.

### 11.2. Impact on Microbial Communities: Disruption of Ecological Balance

The impact of nano-antimicrobials on microbial communities represents one of the most concerning aspects of their environmental release, as these materials can fundamentally alter the structure and function of both environmental and host-associated microbiomes. Laboratory and mesocosm studies consistently demonstrate that nano-antimicrobials can reduce populations of sensitive taxa, shift overall community composition, and alter critical metabolic functions in ways that may cascade through entire ecosystems [156,159,160]. These effects are highly concentration- and material-dependent, varying with exposure duration, nanoparticle type, and the specific characteristics of the microbial community and its environmental matrix.

In aquatic ecosystems, the effects on periphyton and biofilm communities illustrate the potential for nano-antimicrobials to reshape fundamental ecological processes. Chronic exposures to citrate-coated silver nanoparticles at environmentally relevant concentrations (0.1, 1, and 10 μM) led to strong association of silver with periphyton, with 84 to 98% of available silver becoming bound to these biofilm communities [156]. At the highest concentration tested (10 μM), exposures significantly reduced algal primary production and microbial respiration while paradoxically increasing bacterial secondary production and total biomass accrual. This pattern indicates a metabolic shift from autotrophy toward heterotrophy—a fundamental change in ecosystem energy flow that could alter nutrient cycling, oxygen dynamics, and food web structure in affected waters [156]. Such shifts in primary production have implications far beyond the microbial community itself, potentially affecting all organisms that depend on algae as the base of the aquatic food web.

Soil microbial communities, which underpin terrestrial ecosystem functions including nutrient cycling, organic matter decomposition, and plant health, also show significant responses to nano-antimicrobial exposure. Soils exposed to silver nanoparticles exhibited significant taxonomic shifts and altered transcriptional profiles, with upregulation of stress- and metal-response pathways in exposed bacterial communities [160]. These molecular-level responses indicate that even when community-level effects appear subtle, individual populations may be experiencing substantial physiological stress that could affect their ecological roles. The long-term consequences of such chronic stress on soil ecosystem services remain incompletely understood, but the potential for reduced nutrient availability, altered carbon cycling, and decreased soil health represents a significant concern for agricultural and natural terrestrial systems.

### 11.3. Trophic Transfer: Accumulation in the Food Chain

The potential for nano-antimicrobials to enter and accumulate within food webs represents a critical pathway for ecological risk, as biomagnification could expose organisms far removed from initial release points and concentrate toxicity at higher trophic levels. Empirical evidence from both field and laboratory studies demonstrates that nanoparticles can indeed be taken up by primary producers and consumers, with subsequent retention, excretion, or biomagnification patterns that differ markedly depending on the specific material, particle size, and organism involved [157,161]. The complexity of these patterns challenges simple generalizations and highlights the need for material- and ecosystem-specific assessments.

Field studies in natural aquatic ecosystems provide compelling evidence that nano-antimicrobials can bioaccumulate and biomagnify under real-world conditions. Research conducted in Taihu Lake, China, revealed nanoparticulate silver (18.8 to 41.0 nm) in water, sediment, and biota, with a trophic magnification factor of 1.21, indicating that nanoparticulate silver concentrations increased with trophic level [161]. This biomagnification contrasts sharply with the behavior of nanoparticulate titanium (46.6–116 nm) in the same system, which exhibited trophic magnification factor values less than 1, indicating biodilution rather than biomagnification [161]. These divergent patterns demonstrate that different nanomaterials behave very differently in food webs, and that generalizations from one material cannot be reliably applied to others. The Taihu Lake study also identified sediment as a critical exposure reservoir, with benthic organisms serving as important vectors for transferring nanoparticles from sediment into aquatic food webs [161].

Laboratory food chain studies allow controlled investigation of transfer mechanisms and efficiency across trophic links. In a terrestrial system, silver and titanium nanoparticles were transferred from contaminated lettuce to terrestrial snails, with trophic transfer factors for silver ranging from 0.2 to 1.1 and for titanium from 3.8 to 47 [157]. Notably, silver was largely excreted by snails via feces, limiting net accumulation, while titanium tended to accumulate preferentially in the digestive gland [157]. Mixed exposures to both silver and titanium nanoparticles increased biological effects beyond those of single exposures, suggesting potential synergistic toxicity that could be relevant in environmental settings where organisms are exposed to complex mixtures of nanomaterials [157]. These findings illustrate that accumulation is not simply a function of exposure concentration but depends critically on physiological processing, with different metals following distinct uptake, distribution, and elimination pathways.

Aquatic sediment-to-benthic-invertebrate-to-fish food chains have been particularly well studied using stable isotope tracers that allow precise tracking of nanoparticle-derived metals. In experimental systems examining copper oxide nanoparticle transfer from sediment to Tubifex worms to three-spined stickleback fish, sediment-associated copper oxide nanoparticles entered the food web and accumulated in worms at concentrations of approximately 0.7 to 1.1 μg ^65^Cu/g dry weight [162,163]. However, dietary transfer from worms to fish was limited, with only 65 to 80 ng/g dry weight detected in fish intestinal tissue, substantially less than transfer of dissolved copper [162,163]. Substantial egestion of nanoparticle-derived copper by fish indicates that particles or particle-derived metals passing through the digestive system are not efficiently assimilated, limiting biomagnification potential in this particular food chain configuration [162,163]. These controlled studies suggest that while nanoparticles can move across trophic boundaries, efficient assimilation is not guaranteed and varies with the chemical form of the metal and the physiology of the consumer.

### 11.4. Co-Selection for Resistance: A Long-Term Evolutionary Concern

Perhaps the most troubling long-term ecological consequence of environmental nano-antimicrobial pollution is the potential for co-selection of antibiotic resistance, a phenomenon that could undermine the effectiveness of conventional antibiotics that are critical for human and veterinary medicine. Multiple experimental and metagenomic studies demonstrate that sublethal exposures to metal and metal-oxide nanoparticles can increase antibiotic resistance gene abundance and promote mechanisms that facilitate horizontal gene transfer, raising serious concerns about the dissemination of resistance in wastewater, receiving environments, and ultimately in clinical settings [125,164].

Anaerobic digestion systems, which process large volumes of sewage sludge containing concentrated nanoparticles, provide clear evidence of nano-antimicrobial-driven resistance selection. Exposures to copper oxide and zinc oxide nanoparticles during sludge anaerobic digestion increased antibiotic resistance gene abundance without altering the types of resistance genes present, and stimulated signal transduction pathways—particularly 2-component regulatory systems—linked to quorum sensing, pili synthesis, and metal tolerance [125]. These physiological responses correlated with elevated levels of mobile genetic elements and increased antibiotic resistance gene propagation potential, suggesting that nanoparticle stress triggers cellular responses that incidentally facilitate the spread of resistance genes [125]. This finding is particularly concerning because anaerobic digesters process waste from large populations and the resulting biosolids are widely distributed to agricultural lands, potentially spreading resistance genes throughout the environment.

The relationship between nanoparticle exposure and resistance gene selection shows complex dose- and environmental-context dependencies that complicate risk assessment. In estuarine waters, environmentally relevant low doses of zinc oxide nanoparticles (0.2 and 1 mg/L) selected for specific resistance genes including sul1, tetA, ermB, and qnrS, whereas higher doses (10 mg/L) favored different antibiotic resistance gene profiles including sul2 and tetW [164]. These antibiotic resistance gene increases correlated positively with integrase intI1 and transposon markers (Tn916/1545), indicating enhanced horizontal gene transfer capacity [164]. The dose-dependent shift in which resistance genes are selected suggests that different exposure scenarios may favor different resistance profiles, and that the environmental concentration of nano-antimicrobials could shape the specific resistance threats that emerge. Furthermore, salinity and other environmental factors modulated these effects, indicating that the same nanoparticle exposure may produce different resistance outcomes in freshwater versus estuarine versus marine environments [164].

In summary, the environmental nanotoxicology of nano-antimicrobials is governed by their transport, transformation, and interactions within complex environmental matrices. Surface chemistry, aging processes, and co-contaminant interactions significantly influence their fate and ecological impacts. The current body of research underscores the necessity for comprehensive, multidisciplinary approaches to assess and mitigate the ecological risks posed by these emerging nanomaterials. Future research should focus on elucidating long-term transformation pathways, bioaccumulation potential, and the development of predictive models to safeguard ecosystems from unintended adverse effects of nano-antimicrobials.

## 12. The Economic and Manufacturing Horizon: Cost, Scalability, and Global Access

The economic and manufacturing landscape of antimicrobial nanoformulations is a complex domain characterized by challenges related to cost, scalability, and equitable global access. Economic horizon of these technologies—a sector projected to reach USD 5.4 to 8.96 billion by 2033 to 2034, with compound annual growth rates of 11 to 21% across antimicrobial nanomaterials, nanocoatings, and nanomedicine applications [165]. Recent literature underscores the critical need for innovative production methods and sustainable approaches to overcome these barriers, thereby facilitating broader clinical and industrial deployment.

One of the primary concerns in the commercialization of antimicrobial nanoformulations is the high cost associated with their synthesis and manufacturing. As highlighted by the review on multifunctional applications of nanoparticles, despite significant technological advancements, a substantial gap persists in producing multifunctional nanoparticles at scale due to costs and restricted access to fabrication processes [166]. Similarly, the high expenses involved in developing nanotechnology-based healthcare products are recognized as significant barriers, especially in ensuring widespread availability across different regions [167]. The high cost not only limits the affordability of nano-enabled antimicrobials but also hampers their integration into routine clinical practice, which is crucial given the escalating threat of antimicrobial resistance.

Addressing the cost challenge, recent studies advocate for the adoption of scalable and cost-effective synthesis methods. For instance, the mechanochemical approach has been recommended as a promising technique for producing metal oxide nanoparticles, such as copper oxide nanoparticles, in a manner that is both scalable and economical [168]. This method’s automation potential further enhances its suitability for large-scale production, which is essential for meeting global demand. The emphasis on mechanochemical synthesis aligns with the broader goal of reducing manufacturing costs and improving the economic viability of nanoformulations.

Scalability remains a pivotal issue in translating laboratory-scale nanoparticle synthesis into industrial applications. The literature indicates that current manufacturing processes often face limitations in producing nanoparticles at the volume required for widespread use. The review on multifunctional nanoparticles explicitly notes that despite technological progress, issues like uneven manufacturing and restricted scalability hinder the transition from research to real-world applications [166]. To mitigate these challenges, innovative manufacturing strategies such as green nanotechnology are gaining attention. For example, the use of plant-derived nanoparticles offers a sustainable and scalable alternative, leveraging natural resources to produce antimicrobial nanoparticles at lower costs [169]. The environmental benefits and cost-effectiveness of such approaches make them attractive options for large-scale production.

Furthermore, the economic viability of nanotechnology in healthcare is also influenced by regulatory and safety considerations. Ma et al. [167] emphasizes that regulatory frameworks and safety assessments are crucial for ensuring that nanoformulations can be produced and deployed at scale without compromising safety standards. The development of standardized protocols and regulatory support can facilitate smoother pathways from laboratory research to commercial manufacturing, ultimately reducing costs and enhancing global access.

Global access to antimicrobial nanoformulations is a multifaceted issue, compounded by disparities in manufacturing capabilities and regulatory environments across countries. Chaturvedi and Ranjan [170] underscores the importance of equitable distribution and access, especially in regions heavily burdened by antimicrobial resistance. Furthermore, issues like restricted scalability and high prices are major concerns that need to be addressed to ensure broader clinical and industrial feasibility. Strategies such as adopting green synthesis methods, leveraging natural resources, and fostering international collaborations are suggested to bridge these gaps.

In addition, the economic impact of nanotechnology extends beyond manufacturing costs. The potential to combat superbugs and reduce the burden of antimicrobial resistance has significant public health implications. Ho et al. [171] notes that nanotechnology-based antimicrobials could play a vital role in addressing this global health crisis, provided that manufacturing costs are minimized and production processes are scalable. The development of cost-effective, scalable nanomaterials is therefore not only an economic imperative but also a public health priority.

In summary, the current literature emphasizes that overcoming economic and manufacturing barriers is essential for the widespread adoption of antimicrobial nanoformulations. Innovations in synthesis methods, such as mechanochemical and green nanotechnology, offer promising pathways to reduce costs and enhance scalability. Addressing regulatory and safety concerns is equally important to facilitate global access, especially in resource-limited settings. As antimicrobial resistance continues to threaten global health, the strategic focus on cost-effective, scalable, and sustainable nanomanufacturing will be pivotal in translating laboratory breakthroughs into accessible, effective antimicrobial therapies worldwide.

## 13. Limitations and Future Prospects

The multifaceted mechanistic actions of antimicrobial nanoformulations present both promising opportunities and notable limitations. These nanoformulations, which include nanoparticles such as silver, transition metals, and other nanomaterials, offer diverse mechanisms to combat bacterial infections, including oxidative stress induction, metal ion release, and membrane disruption. However, challenges such as potential cytotoxicity, environmental impact, and the complexity of regulatory approval processes limit their widespread application. Future prospects focus on enhancing the efficacy and safety of these nanoformulations through innovative delivery systems and combinatorial approaches.

- Incomplete in vivo mechanistic validation: Many studies rely heavily on in vitro assays with limited in vivo validation of antimicrobial mechanisms of nanoformulations. Systematic in vivo studies should be conducted to elucidate molecular pathways and host–pathogen–nanomaterial interactions, focusing on reactive oxygen species generation, membrane disruption, and intracellular effects. In vivo validation is essential to confirm efficacy and safety, as in vitro results may not fully represent complex biological environments [172,173,174].
- Standardization of synergistic combination protocols: Variability in nanoparticle synthesis, antibiotic loading, and bacterial strains complicates comparison of synergistic effects. Standardized protocols should be developed for nanoparticle-antibiotic combination studies, including uniform characterization, dosing regimens, and resistance profiling. Standardization will enable reproducibility, facilitate meta-analyses, and optimize clinical translation of synergistic therapies [99,123].
- Limited clinical translation and scalability data: Few nanoformulations have progressed to clinical trials; challenges in scalable production and regulatory approval persist. Scalable, cost-effective synthesis methods should be investigated and regulatory guidelines should be established; clinical trials should be conducted to assess long-term safety and efficacy. Addressing manufacturing and regulatory barriers is critical for transitioning nanoformulations from bench to bedside [101,172,175].
- Insufficient long-term biosafety and toxicity profiling: Long-term toxicity, immune responses, and microbiota impacts of nanoformulations remain underexplored. Comprehensive chronic toxicity and immunogenicity studies should be performed in relevant animal models; microbiome alterations should be assessed post-treatment. Safety concerns limit clinical adoption; thorough biosafety data are needed to ensure patient safety and regulatory compliance [172,173,174].
- Mechanistic understanding of bacterial resistance to nanoparticles: Emerging evidence shows bacteria can develop resistance mechanisms against nanoparticles, such as efflux pumps and genetic adaptations. Molecular mechanisms of nanoparticle resistance development should be investigated; nanoparticles should be designed to circumvent or inhibit resistance pathways like efflux pumps. Understanding resistance evolution is vital to sustain long-term efficacy of nano-antimicrobials and prevent resistance emergence [123,124,126].
- Optimization of targeted delivery and biofilm penetration: Variability exists in nanoparticle penetration efficiency into biofilms and intracellular pathogens; delivery platforms are often in early development. Advanced surface functionalization and stimuli-responsive delivery systems should be developed and tested to enhance biofilm and intracellular targeting in vivo. Effective targeting is crucial for treating chronic and resistant infections associated with biofilms and intracellular reservoirs [176,177].
- Lack of comprehensive pharmacokinetics and pharmacodynamics data: Pharmacokinetic and pharmacodynamic profiles of many nanoformulations are poorly characterized. Detailed pharmacokinetics and pharmacodynamics studies should be conducted to understand biodistribution, clearance, and therapeutic windows of nanoformulations in animal models and humans. Pharmacokinetics and pharmacodynamics data are essential for dose optimization, minimizing toxicity, and maximizing therapeutic efficacy [123,178,179].
- Limited exploration of green synthesis impact on efficacy and safety: Green synthesis methods show promise in reducing toxicity but their influence on antimicrobial efficacy and reproducibility is not fully understood. Green-synthesized nanoparticles should be systematically compared with conventional ones regarding antimicrobial activity, stability, and biocompatibility. Green synthesis may offer sustainable, safer alternatives but requires rigorous evaluation to ensure clinical viability [180,181].
- Understudied interactions with human microbiota: The bidirectional interactions between nanoparticles and human microbiota are poorly characterized. How nanoformulations affect commensal microbiota composition and function should be investigated; microbiota-informed design strategies should be designed for safe nanomedicines. Microbiota perturbations can influence host immunity and treatment outcomes; understanding these interactions is critical for safe therapy [172].
- Insufficient mechanistic clarity on multi-target and stimuli-responsive nanoparticles: The precise molecular mechanisms and efficacy of stimuli-responsive and multifunctional nanoparticles remain inadequately elucidated. Mechanistic studies combining molecular biology and advanced imaging should be performed to clarify how stimuli-responsive nanoparticles exert antimicrobial effects under physiological conditions. Detailed mechanistic insights will guide rational design of next-generation intelligent nanomedicines with enhanced specificity and reduced side effects [182,183].

While the potential of antimicrobial nanoformulations is significant, it is crucial to address the limitations associated with their use. The development of safe and effective nanoformulations requires interdisciplinary collaboration and rigorous research to overcome challenges related to toxicity, resistance, and regulatory approval. By focusing on these areas, the field can advance towards the successful clinical translation of these promising technologies, ultimately contributing to the global effort to combat antimicrobial resistance.

## 14. Conclusive Remarks

The current literature presents a compelling overview that antimicrobial nanoformulations offer multifaceted mechanistic pathways to effectively combat antimicrobial resistance pathogens. These nanoformulations leverage diverse modes of action including membrane disruption, reactive oxygen species generation, interference with nucleic acids and proteins, enzymatic inhibition, and efflux pump suppression. The simultaneous targeting of multiple bacterial components is a distinctive advantage, lowering the risk of resistance evolution and enhancing antimicrobial potency. Furthermore, stimuli-responsive and bioengineered nanomaterials demonstrate adaptive antimicrobial actions that improve specificity and minimize off-target effects, emphasizing the versatility of these platforms.

Synergistic enhancement of conventional antibiotics by nanoformulations is widely reported, where nanoparticles facilitate improved drug delivery, reduced minimum inhibitory concentrations, and restoration of antibiotic efficacy against resistant strains. Synergy often arises from nanoparticles’ abilities to disrupt biofilms, inhibit resistance mechanisms such as efflux pumps, and promote intracellular antibiotic accumulation. These combinational approaches appear to be a promising strategy to revitalize existing antibiotics and extend their clinical utility.

Targeting of biofilms and intracellular pathogens is a critical strength of nanoformulations. Nanoparticles’ tunable size, surface charge, and functionalization enable efficient biofilm penetration and disruption, overcoming one of the major barriers in chronic infections. Delivery platforms based on lipids, polymers, and hybrid systems further enhance targeting capabilities, facilitating controlled release and reducing toxicity. However, in vivo validation of biofilm and intracellular targeting remains limited, highlighting a need for more translational studies.

Safety and biocompatibility are variable across nanomaterial classes, with polymeric and lipid-based nanoparticles generally exhibiting favorable profiles. Green synthesis and surface functionalization techniques contribute to reduced cytotoxicity and improved host compatibility. Nevertheless, comprehensive and long-term toxicity assessments, immunological responses, and environmental impact evaluations are essential prerequisites for clinical translation.

Translational readiness has advanced with several clinical trials underway, yet challenges persist in scaling up manufacturing, achieving regulatory approval, ensuring reproducibility, and maintaining product stability. Complex nanocarrier designs face hurdles in large-scale production and standardization. Additionally, emerging evidence of bacterial adaptation to nanoparticles underscores the necessity for vigilant resistance monitoring.

In conclusion, antimicrobial nanoformulations represent a transformative and promising approach to overcoming antimicrobial resistance by integrating multifaceted antimicrobial mechanisms, synergistic antibiotic enhancement, and targeted delivery strategies. To fully realize their clinical potential, future research should focus on standardized in vivo studies, detailed safety profiling, scalable manufacturing processes, and regulatory harmonization. Such efforts will pave the way for effective, safe, and sustainable nanomedicine-based antimicrobial therapies.

## Figures and Tables

**Table 1:** Current therapeutic formulations targeting bacterial biofilms: mechanisms of action and efficacy reported in literature.

Strategy	Formulation	Target	Proposed Mechanism of Anti-Biofilm Action	Ref.
Antibiotic therapy	Colistin	*P. aeruginosa*	Concentration-dependent killing; efficacy linked to AUC/MBIC ratio for biofilm eradication in vivo.	[59]
	Azithromycin	*P. aeruginosa*	Inhibits quorum sensing, reducing virulence factor production and biofilm formation.	[60]
Combination therapy	Tobramycin + Aztreonam	*P. aeruginosa*	Alternating use enhances biomass reduction and lowers resistance risk via antagonistic resistance mechanisms.	[61]
	Penicillin + Gentamicin	*S. aureus*, *E. coli*	Dual action: inhibits cell wall synthesis (Penicillin) and protein synthesis (Gentamicin).	[62]
	Gentamicin + Melittin	Methicillin-resistant *S. aureus*, *P. aeruginosa*	Melittin disrupts biofilm structure, enhancing Gentamicin penetration and permeability.	[63]
	Azithromycin + Nisin	Methicillin-resistant *S. aureus*	Synergy inhibits growth/attachment and reduces expression of resistance genes.	[64]
Surface coatings	Antibiotic coatings (e.g., on implants)	Methicillin-resistant *S. aureus*, *P. aeruginosa*	Prevents bacterial adhesion and provides sustained local antibiotic release.	[65]
	Antimicrobial peptide coatings	Methicillin-resistant *S. aureus*, *S. mutans*	Direct membrane disruption or inhibition of biofilm formation on the coated surface.	[66]
	Metal ion coatings (e.g., silver, zinc)	*S. aureus*, *P. aeruginosa*	Released ions disrupt bacterial membranes, metabolism, and redox balance.	[67]
	Antibacterial polymer coatings	*S. aureus*, *P. aeruginosa*	Creates anti-fouling surfaces or releases embedded antimicrobial agents.	[68]
Small molecule inhibitors	Generic anti-biofilm compound	*P. aeruginosa*	Reduces intracellular c-di-GMP levels, a key regulator of biofilm formation.	[69]
	5-benzylidene-4-oxazolidinones	Methicillin-resistant *S. aureus*	Dose-dependent inhibition of bacterial attachment and biofilm structural integrity.	[70]
Enzyme therapy	Bovine microbial enzyme	*K. pneumoniae*	Degrades biofilm matrix polysaccharides (e.g., glycan).	[71]
	Serratiopeptidase	*K. pneumoniae*	Degrades protein and polysaccharide components of the biofilm matrix.	[72]
Immunotherapy	EbpA vaccine	*Enterococcus faecalis*	Blocks bacterial attachment to fibrinogen-coated surfaces (e.g., catheters).	[73]

**Table 2:** Clinical trials of nanoformulations based anti-biofilm treatments [58,75].

Trade Name (or Identifier)	Nanoformulation	Antimicrobial Agent	Target Pathogen	Clinical Trial	Clinical Trial Number
Ferumoxytol	Iron oxide nanoparticles	Sodium hypochlorite	Apical periodontitis	Phase IV	NCT06110494
Pulmaquin	Liposome	Ciprofloxacin	*P. aeruginosa* (in non-cystic fibrosis bronchiectasis)	Phase III	NCT02104245, NCT01515007
Arikace	Liposome	Amikacin	*P. aeruginosa* (in cystic fibrosis)	Phase III	NCT01315691, NCT01316276
AgTive (Logicath)	Silver nanoparticles	Silver	Central venous catheter-related infections/bacteremia	Phase IV	NCT00337714
Silvasorb	Silver nanoparticles	Silver	Topical infection (in patients with hemiparesis)	Phase III	NCT00659204
-	Silver/copper nanoparticles	Silver and Copper	*S. aureus*/*P. aeruginosa*	-	NCT04775238
-	Polymeric nanoparticles	Ciprofloxacin	*E. faecalis* (root canal infection)	Phase I	NCT05442736
-	Silver nanoparticles	Silver	*S. mutans*/dental caries	Phase III	NCT05221749
-	Polymeric nanoparticles	Doxycycline	Chronic periodontitis (*Porphyromonas gingivalis*)	Phase II	NCT02726646
-	Polymeric nanoparticles	Ammonium polyethyleneimine	*E. faecalis* (root canal infection)	Phase II	NCT01167985

## Data Availability

No new data were created or analyzed in this study. Data sharing is not applicable to this article.
